# Magmatic evolution of Panama Canal volcanic rocks: A record of arc processes and tectonic change

**DOI:** 10.1371/journal.pone.0176010

**Published:** 2017-05-10

**Authors:** David W. Farris, Agustin Cardona, Camilo Montes, David Foster, Carlos Jaramillo

**Affiliations:** 1 Florida State University, Department of Earth, Ocean, and Atmospheric Sciences, Tallahassee, Florida, United States of America; 2 Universidad Nacional de Colombia, Medellín, Colombia; 3 Yachay Tech University, Department of Geological Sciences, San José de Urcuquí, Ecuador; 4 University of Florida, Department of Geological Sciences, Gainesville, Florida, United States of America; 5 Smithsonian Tropical Research Institute, Unit 0948, APO AA, United States of America; Cardiff University, UNITED KINGDOM

## Abstract

Volcanic rocks along the Panama Canal present a world-class opportunity to examine the relationship between arc magmatism, tectonic forcing, wet and dry magmas, and volcanic structures. Major and trace element geochemistry of Canal volcanic rocks indicate a significant petrologic transition at 21–25 Ma. Oligocene Bas Obispo Fm. rocks have large negative Nb-Ta anomalies, low HREE, fluid mobile element enrichments, a THI of 0.88, and a H_2_O_calc_ of >3 wt. %. In contrast, the Miocene Pedro Miguel and Late Basalt Fm. exhibit reduced Nb-Ta anomalies, flattened REE curves, depleted fluid mobile elements, a THI of 1.45, a H_2_O_calc_ of <1 wt. %, and plot in mid-ocean ridge/back-arc basin fields. Geochemical modeling of Miocene rocks indicates 0.5–0.1 kbar crystallization depths of hot (1100–1190°C) magmas in which most compositional diversity can be explained by fractional crystallization (F = 0.5). However, the most silicic lavas (Las Cascadas Fm.) require an additional mechanism, and assimilation-fractional-crystallization can reproduce observed compositions at reasonable melt fractions. The Canal volcanic rocks, therefore, change from hydrous basaltic pyroclastic deposits typical of mantle-wedge-derived magmas, to hot, dry bi-modal magmatism at the Oligocene-Miocene boundary. We suggest the primary reason for the change is onset of arc perpendicular extension localized to central Panama. High-resolution mapping along the Panama Canal has revealed a sequence of inward dipping maar-diatreme pyroclastic pipes, large basaltic sills, and bedded silicic ignimbrites and tuff deposits. These volcanic bodies intrude into the sedimentary Canal Basin and are cut by normal and subsequently strike-slip faults. Such pyroclastic pipes and basaltic sills are most common in extensional arc and large igneous province environments. Overall, the change in volcanic edifice form and geochemistry are related to onset of arc perpendicular extension, and are consistent with the idea that Panama arc crust fractured during collision with South America forming the observed Canal extensional zone.

## Introduction

Volcanic arcs are one of the primary loci of magmatic evolution on Earth, and play a critical role in the compositional development of continental crust (e.g. [1, 2, 3, 4, 5]). In addition, arcs and subduction zones are complex geochemical systems that integrate magmatic and fluid components from the mantle, crust and subducting slab. Determining the relative importance of each of these inputs is often ambiguous. However, a general consensus exists that most arc magmatism results from the dehydration of hydrous minerals (e.g. amphibole, chlorite, antigorite, phlogopite [6, 7] within the subducting slab at depths between 75–170 km [8, 9]. Fluids from the slab flux the overlying mantle wedge and induce partial melting leading to the production of large ion lithophile (LILE) enriched and high-field strength element (HFSE) depleted basaltic magmas [10, 7]. Such hydrous magmas then ascend, pond in the mid-crust to form plutons, and erupt on the surface to form arc volcanoes. This view of arc magmatism can explain much of the generalized geochemical characteristics we observe in arc volcanic and plutonic rocks, however it is essentially a static view how arcs operate. In reality, almost all arcs exhibit significant temporal and spatial variability, and understanding what causes such variability is an important frontier in the comprehension of arc physical and chemical processes [11, 12, 13, 14, 15].

Our general strategy for understanding this problem is to examine arcs that have complex, but understandable histories and to examine how they evolve over time. In this contribution, we focus on evolution of the Panama magmatic arc. We examine the geochemical and tectonic changes that occurred at the Oligocene-Miocene boundary that Farris et al. [16] correlates with the onset of collision between the Panama Block and South America. A key aspect of the Panama arc is that it has a distinct beginning. It initiated at 73–75 Ma atop the trailing edge of the Caribbean plate [17]. This initiation point provides a limit on potential complexities particularly in comparison to Cordilleran arcs in both North and South America that have magmatic histories extending back hundreds of millions of years. However, it also lies at what is now the intersection of five plates and crustal blocks and so there are multiple potential events that have occurred during its evolution.

Wörner et al. [18], Wegner et al. [19] and Farris et al. [16] divide Panama arc activity into an initial depleted arc and an enriched Miocene arc. Wörner et al. [18] and Wegner et al. [19] grouped the Canal volcanic rocks into the enriched Miocene arc. Whattam et al. [20], has also proposed the existence of an adakite-like group of 25–30 Ma volcanic rocks immediately to the west and east of the Canal region that may have formed via a slab tear. Farris et al. [16] showed that the Miocene Canal rocks form a unique group that are depleted in fluid mobile elements and have extensional characteristics that result from fracturing of the Isthmus of Panama lithosphere during initial collision with South America. The end of the Miocene arc in Panama is not well defined, although modern magmatism exists only west of the Canal region and is characterized by adakite-like magmatism in rocks younger than 2–3 Ma. Panama adakites have been variously ascribed to slab-melting [21, 22, 23, 20], a slab-window [24], subduction erosion [25], multiple stage fraction processes [26], or oblique subduction [27]. Overall, our goal is to present a high-resolution picture of the petrologic, volcanologic and structural evolution of the rocks along the Panama Canal and to determine how onset of the Panama block-South America collision influences arc processes.

## Observations and data

### Geologic units, stratigraphy and structural mapping

**Fig 1:**
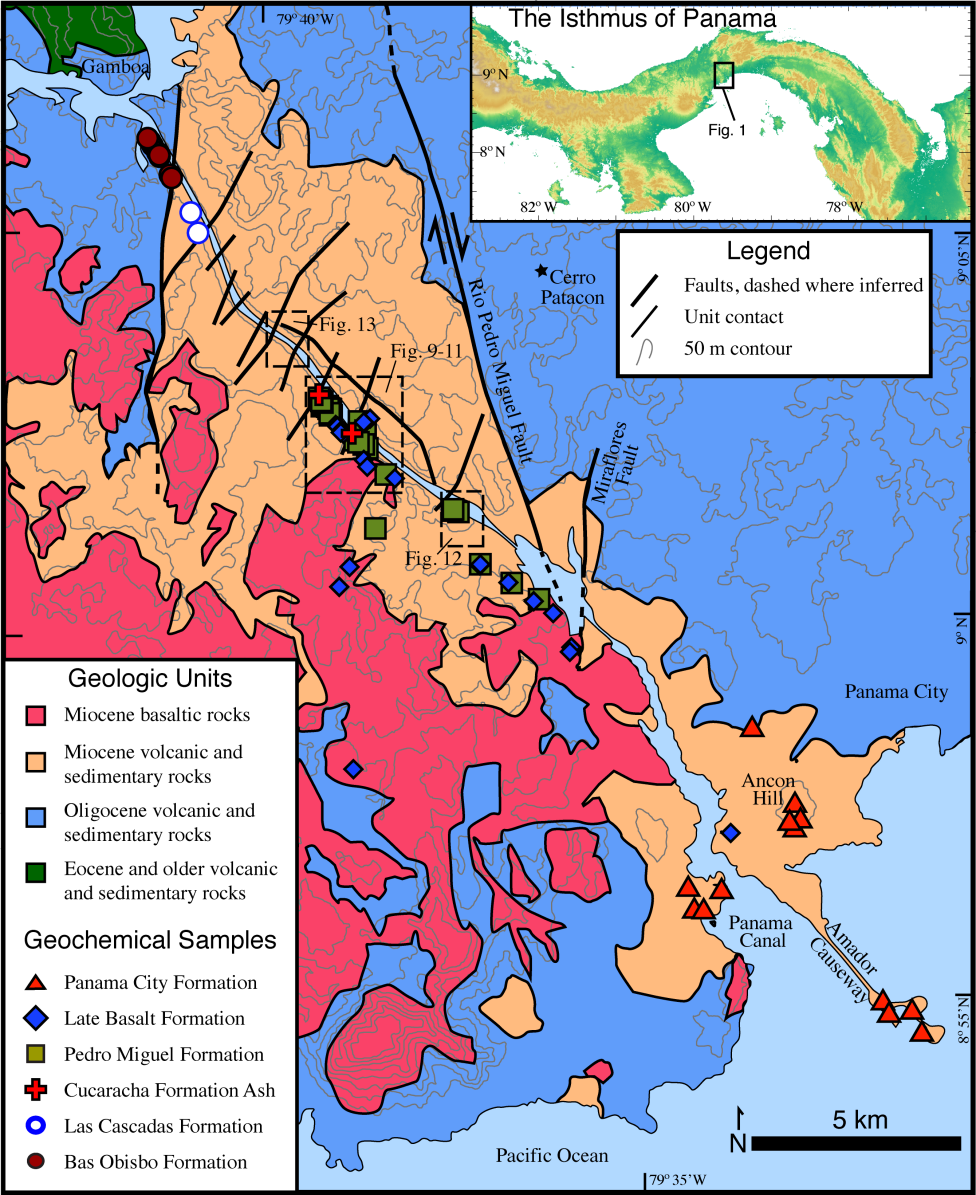
Geologic map of the southern Panama Canal basin adapted from Stewart and Stewart [30]. Symbols indicate the location of geochemical samples. Boxes denote the location of high resolution mapping areas.

**Fig 2:**
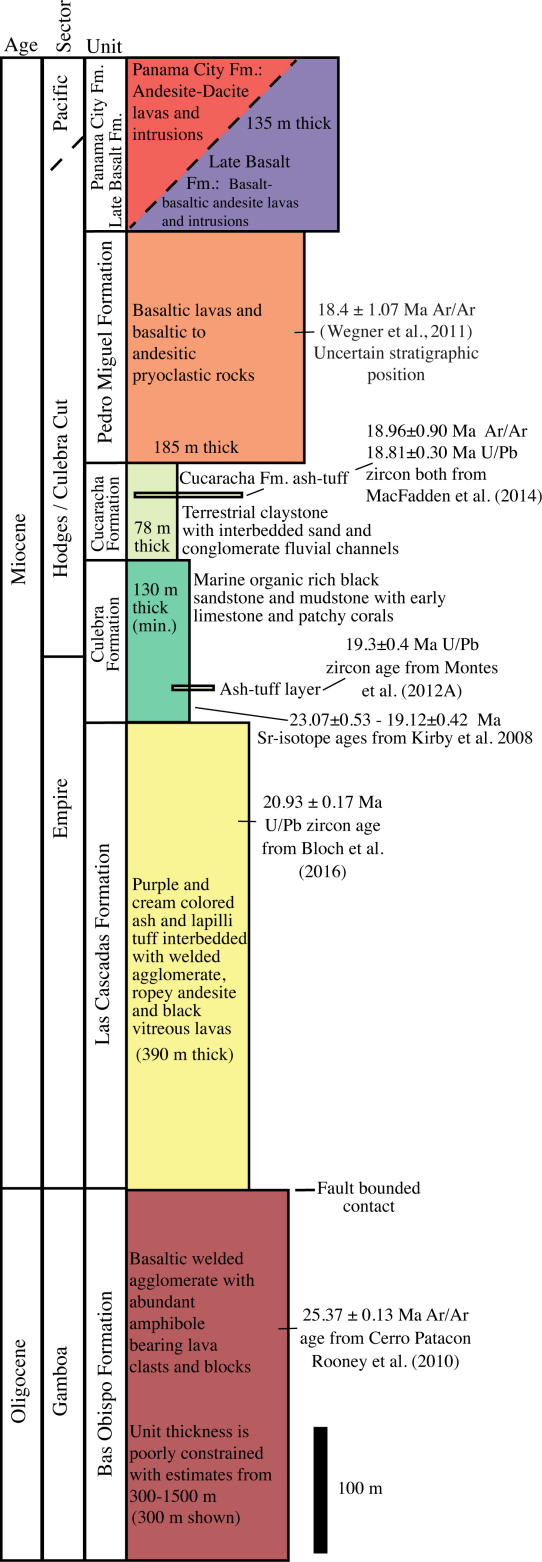
Stratigraphic column of volcanic and sedimentary rocks exposed along the southern Panama Canal. Depicted total thickness of section is 1218 m. Unit thicknesses are from this study, Montes et al. [33], Kirby et al. [31], and Lutton and Banks [29]. Most units have lateral variations in thickness and structural complications that make absolute thickness determinations difficult. Therefore minimum well-constrained unit thicknesses are shown.

**Fig 3:**
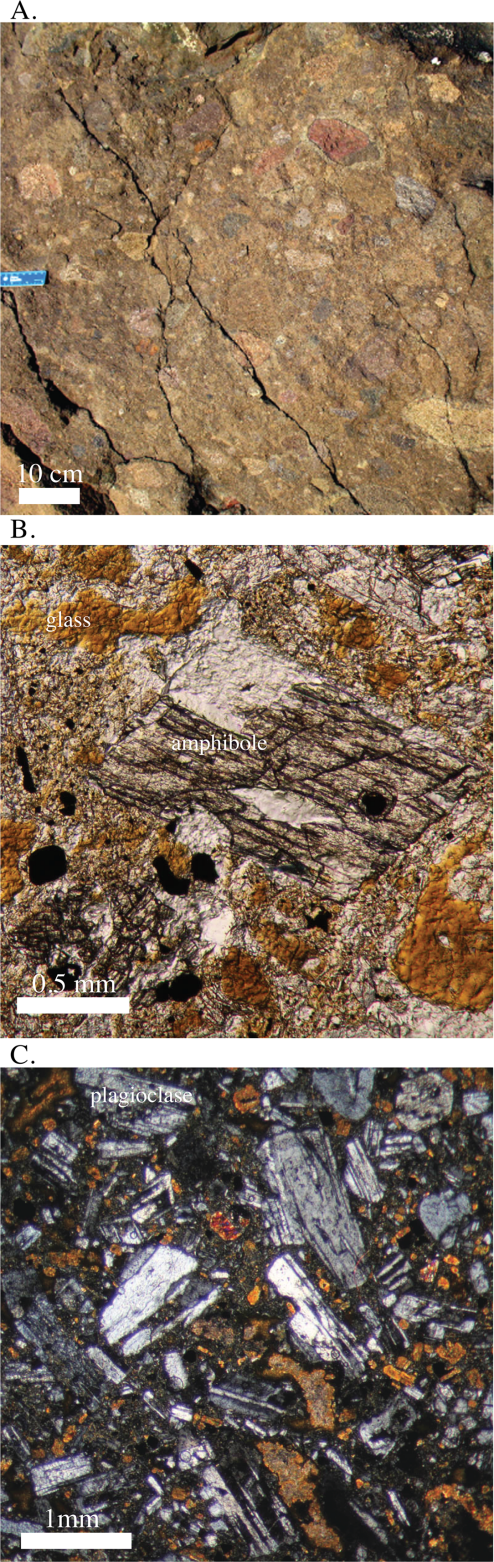
Photos and photomicrographs of the Bas Obispo Formation. A) Field photo of the welded basaltic pyroclastic rocks characteristic of the Bas Obispo Formation. B) Photomicrograph of an amphibole phenocryst surrounded by basaltic glass. C) Photomicrograph of plagioclase phenocrysts surrounded by pyroclastic fragments and pockets of basaltic glass.

**Fig 4:**
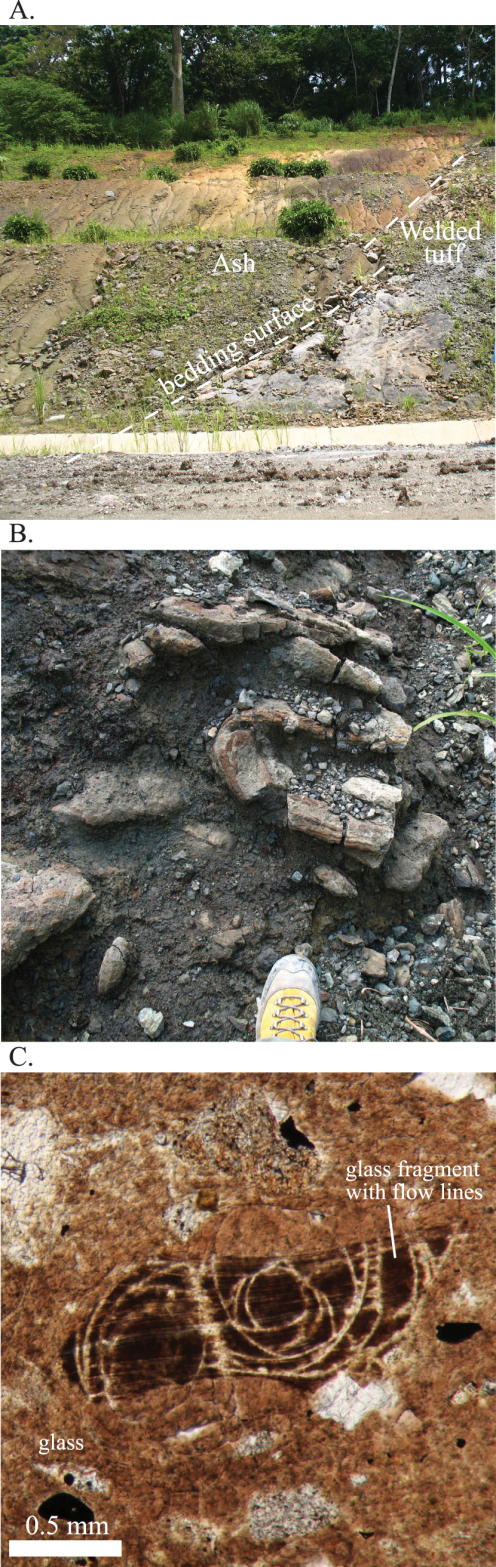
Photos and photomicrographs of the Las Cascadas Formation. A) Field photo of the contact between a welded silicic pyroclastic bed and an ash fall tuff layer typical of the Las Cascadas Formation. B) Ropey andesitic lava C. Photomicrograph of dacitic obsidian layer. Note glass fragment with flow lines surrounded by amorphous glass.

**Fig 5:**
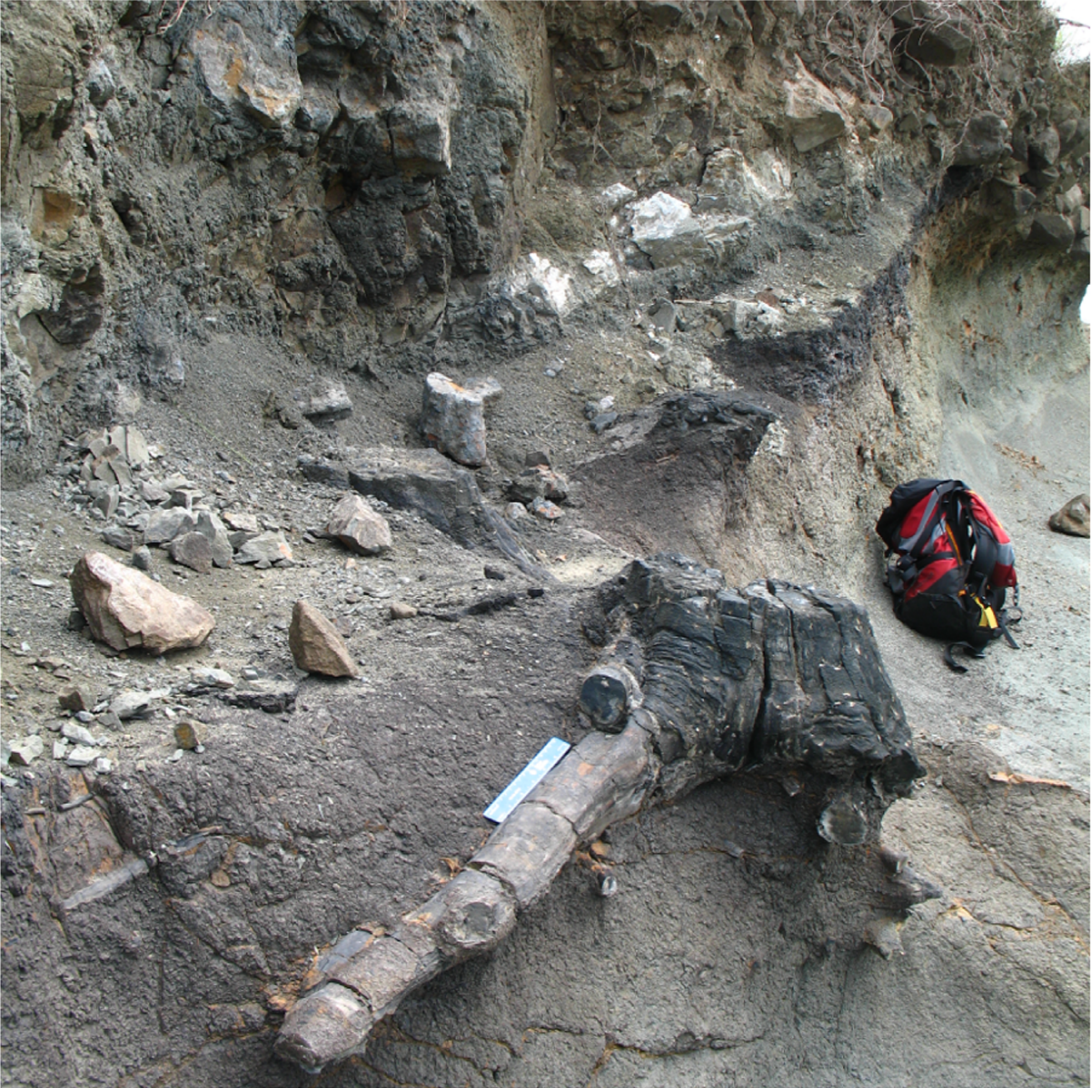
Field photo of a fossilized tree trunk within the Cucaracha Formation ash fall tuff.

**Fig 6:**
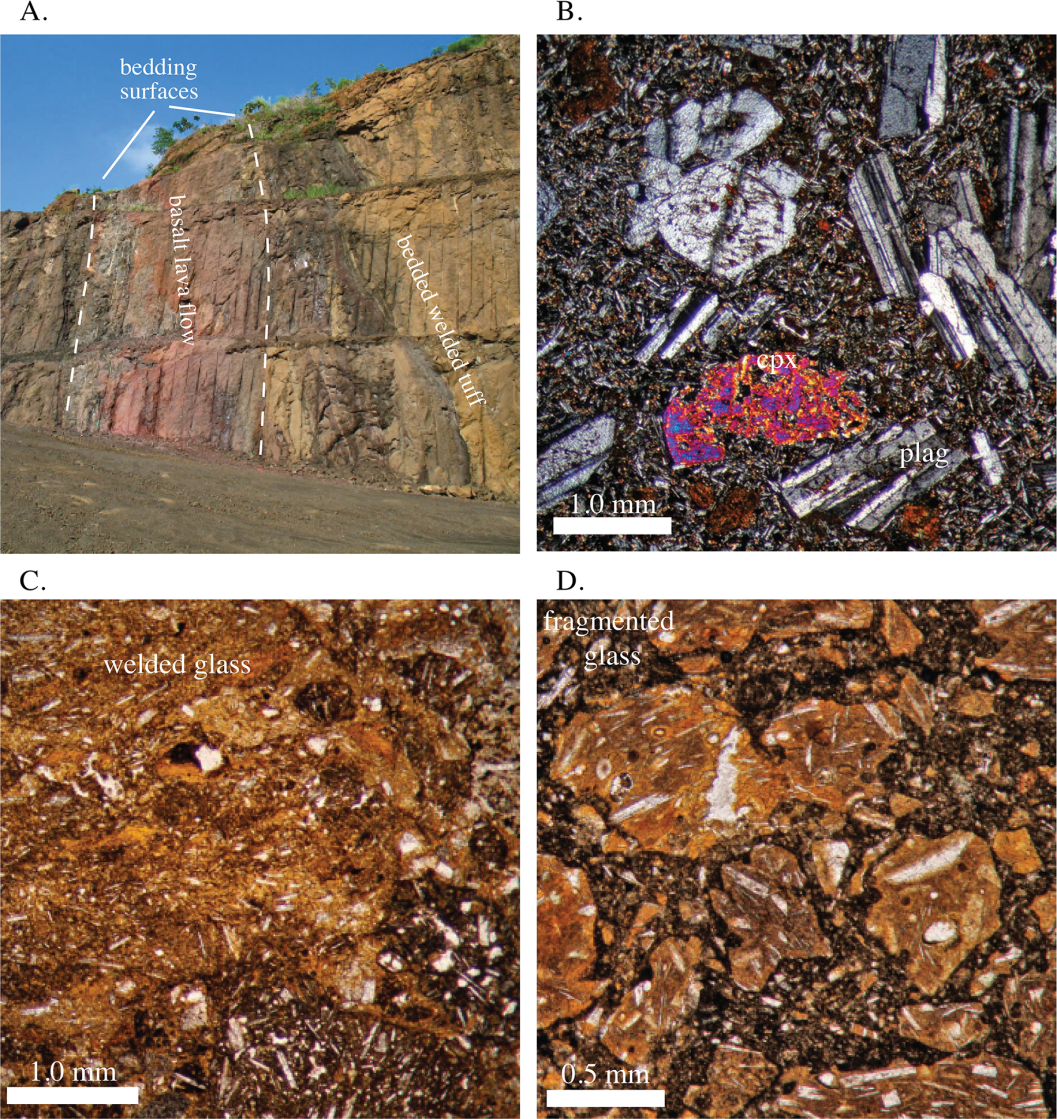
Field photos and photomicrographs of the Pedro Miguel Formation. A) Field photograph of inward dipping basaltic lava flows and welded pyroclastic deposits. B) Photomicrograph of a Pedro Miguel lava flow. Note the equal sized sub-to-euhedral clinopyroxene and plagioclase phenocrysts surrounded by plagioclase microlites. C) Photomicrograph of welded pyroclastic deposit. Note stretched glass bands. D) Photomicrograph of fragmented pyroclastic deposit. Note angular welded pyroclastic fragments surrounded by darker fine-grained matrix.

**Fig 7:**
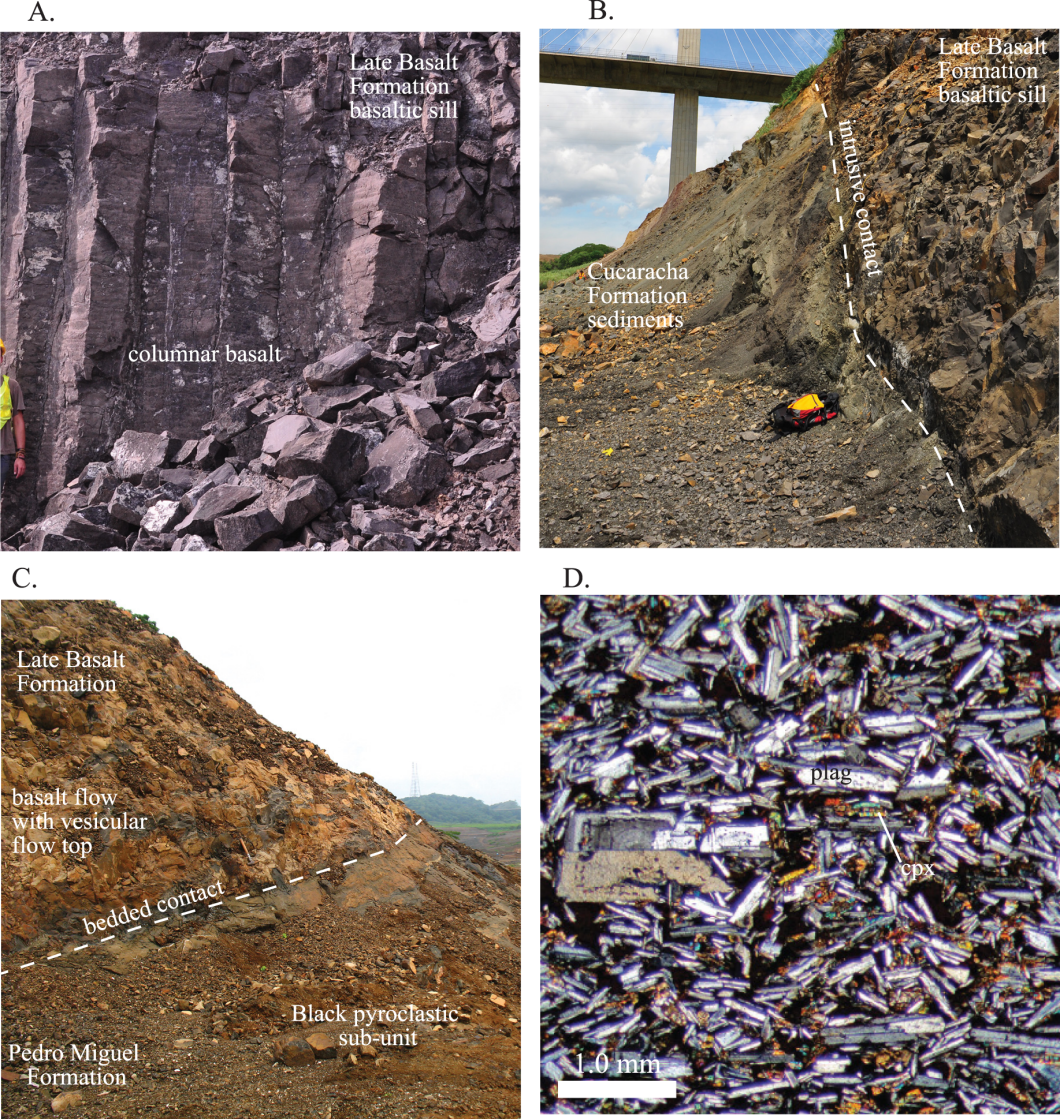
Field photographs and photomicrographs of the Late Basalt Formation. A) Columnar basalts within a large sill. B) Near vertical marginal contact between a Late Basalt sill and the Cucaracha Formation. C) A stratigraphic contact between underlying Pedro Miguel Formation pyroclastic deposits and overlying Late Basalt Formation lava flows. D) Photomicrograph from a Late Basalt Formation sill. The strong plagioclase alignment and trachytic texture is characteristic. Also note the interstitial anhedral clinopyroxene.

**Fig 8:**
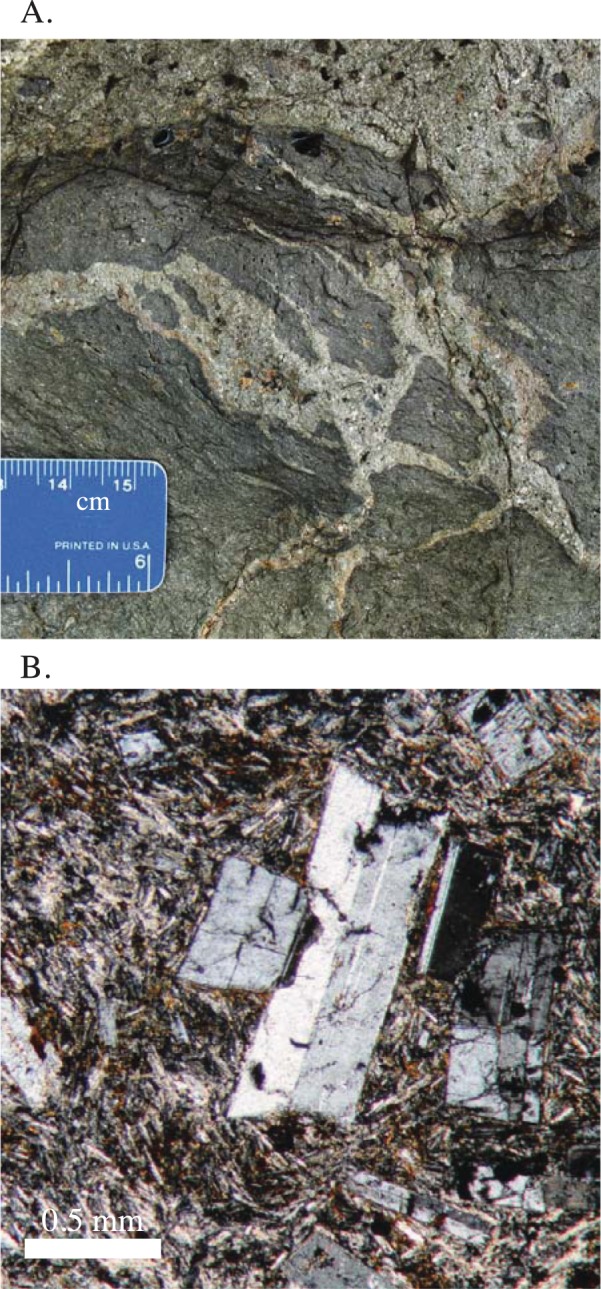
Field photographs and photomicrographs of the Panama City Formation. A) Field photograph of the intermingling of dacitic and andesitic magmas within a shallow intrusive body. B) Euhedral plagioclase phenocrysts surrounded by microlites from the Ancon Hill dacitic plug.

**Fig 9:**
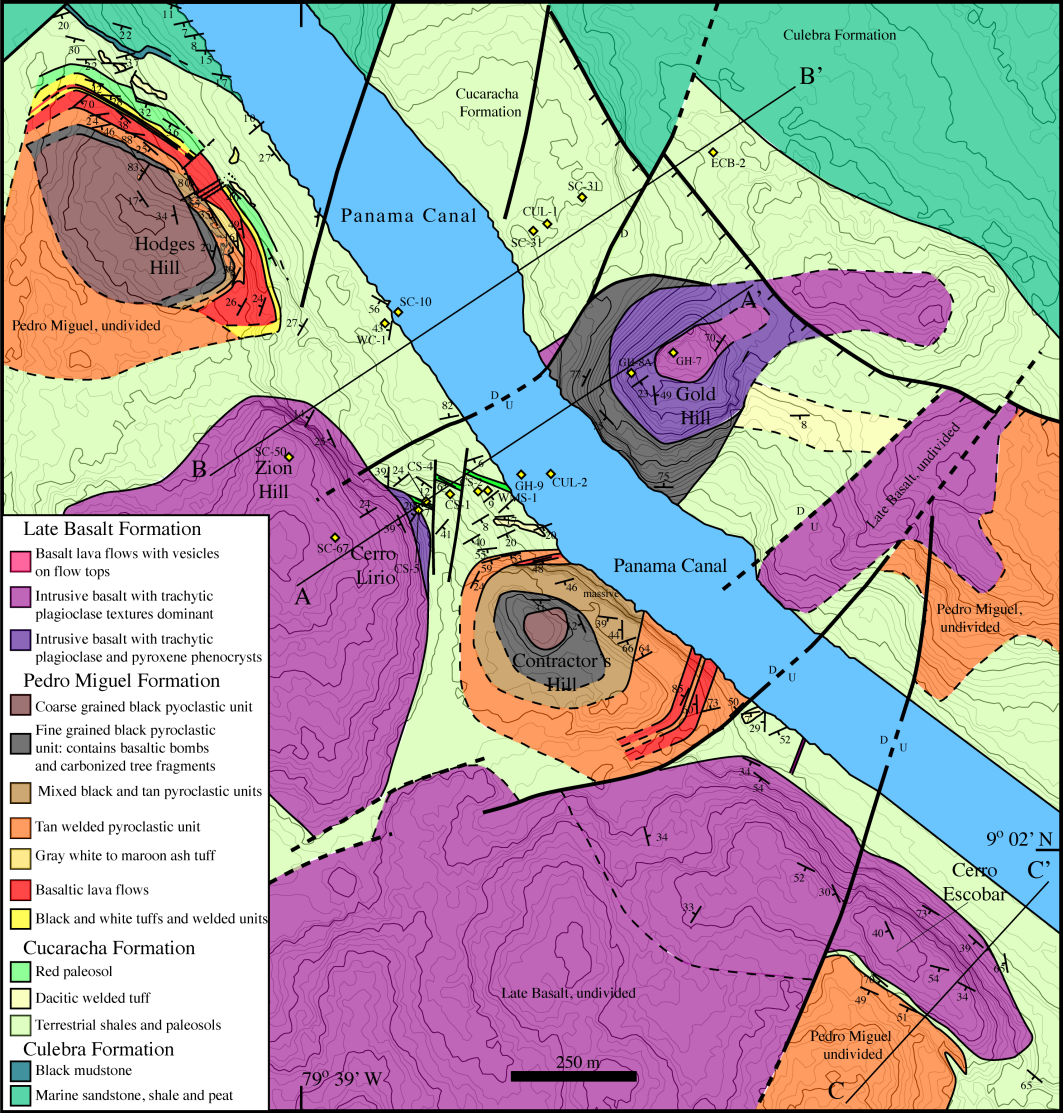
Geologic map of the Culebra Cut along the Panama Canal. The map depicts Miocene pyroclastic pipes of the Pedro Miguel Formation, and large basaltic sills and flows of the Late Basalt Formation both of which intrude and are deposited atop the sedimentary Cucaracha Formation. See Fig 1 for map location.

**Fig 10:**
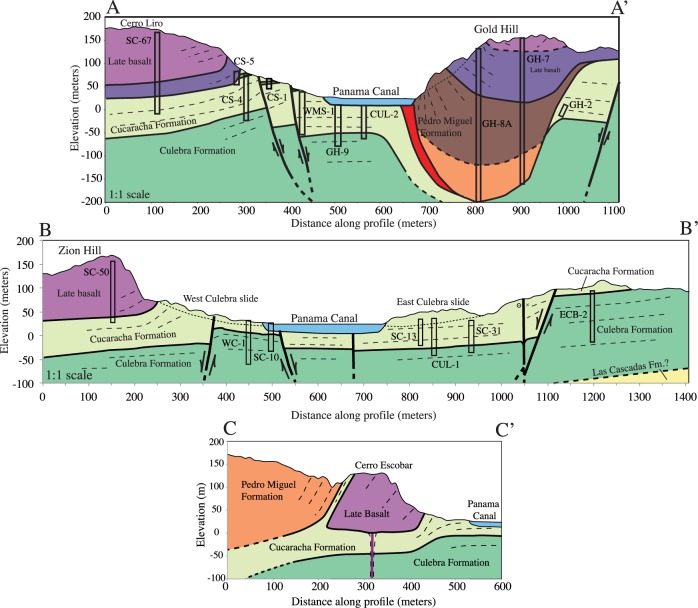
Geologic cross-sections A-A’, B-B’, and C-C’ across the Culebra Cut. See Fig 9. for line locations. Cross-sections are derived from structural field observations and constrained by drill core data from Lutton and Banks [29]. These cross-sections clearly show the subsurface thickness of basaltic sills and pyroclastic bodies and provide vertical offset constraints for normal faults. The cross-sections indicate that the Panama Canal is roughly centered on a small structural graben.

**Fig 11:**
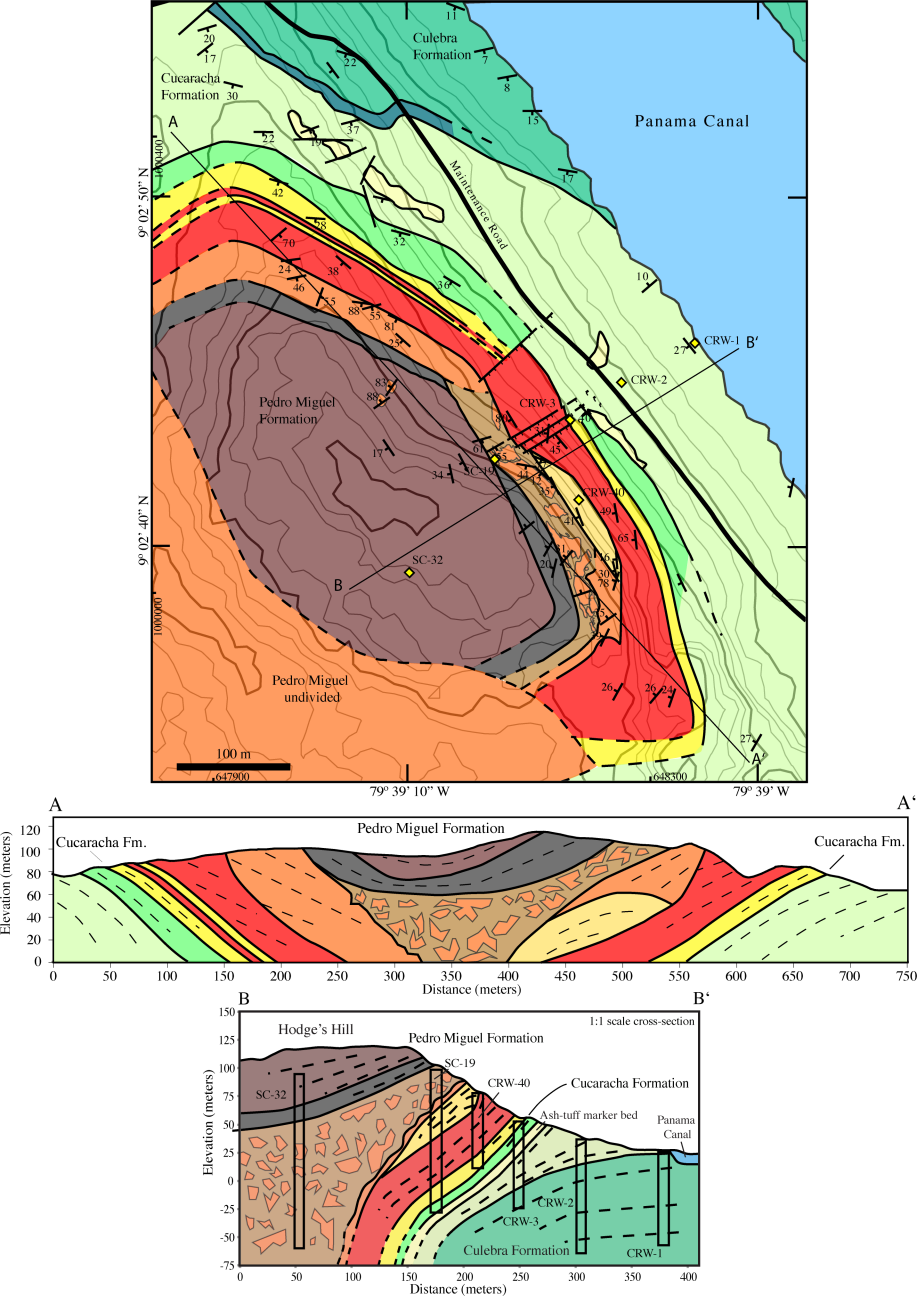
Geologic map and cross-section of Hodges Hill. Hodges Hill is the “type” Pedro Miguel Formation locality. It is composed of inward dipping pyroclastic deposits and lava flows deposited over multiple episodes of explosive eruptions. The 3-D internal geometry as shown in cross-sections A-A’ and B-B’ are derived from structural measurements and Lutton and Banks [29] drill core data. See Fig 9 for unit key.

**Fig 12:**
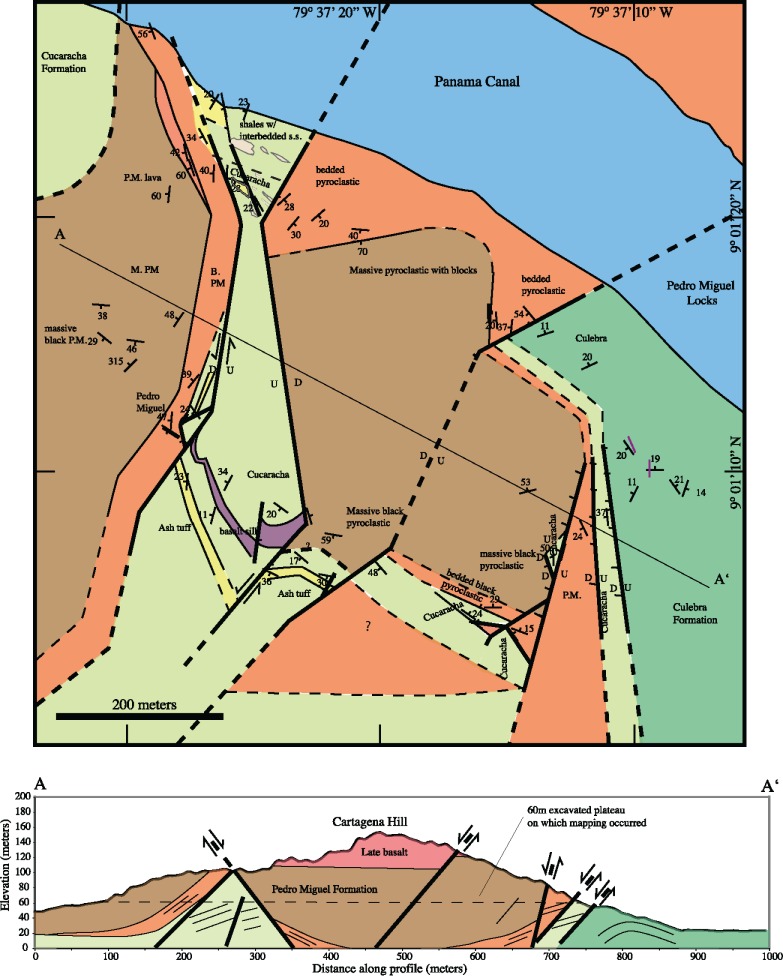
Cartagena Hill geologic map and cross-section. At this locality, inward dipping Pedro Miguel Formation pyroclastic strata are observed, but the volcanic edifice is partially dismembered by subsequent normal and strike slip faulting. Cross-section A-A’ shows pre-excavation topography. The horizontal dashed line on the cross-section indicates the excavation level on which geologic mapping was conducted.

**Fig 13:**
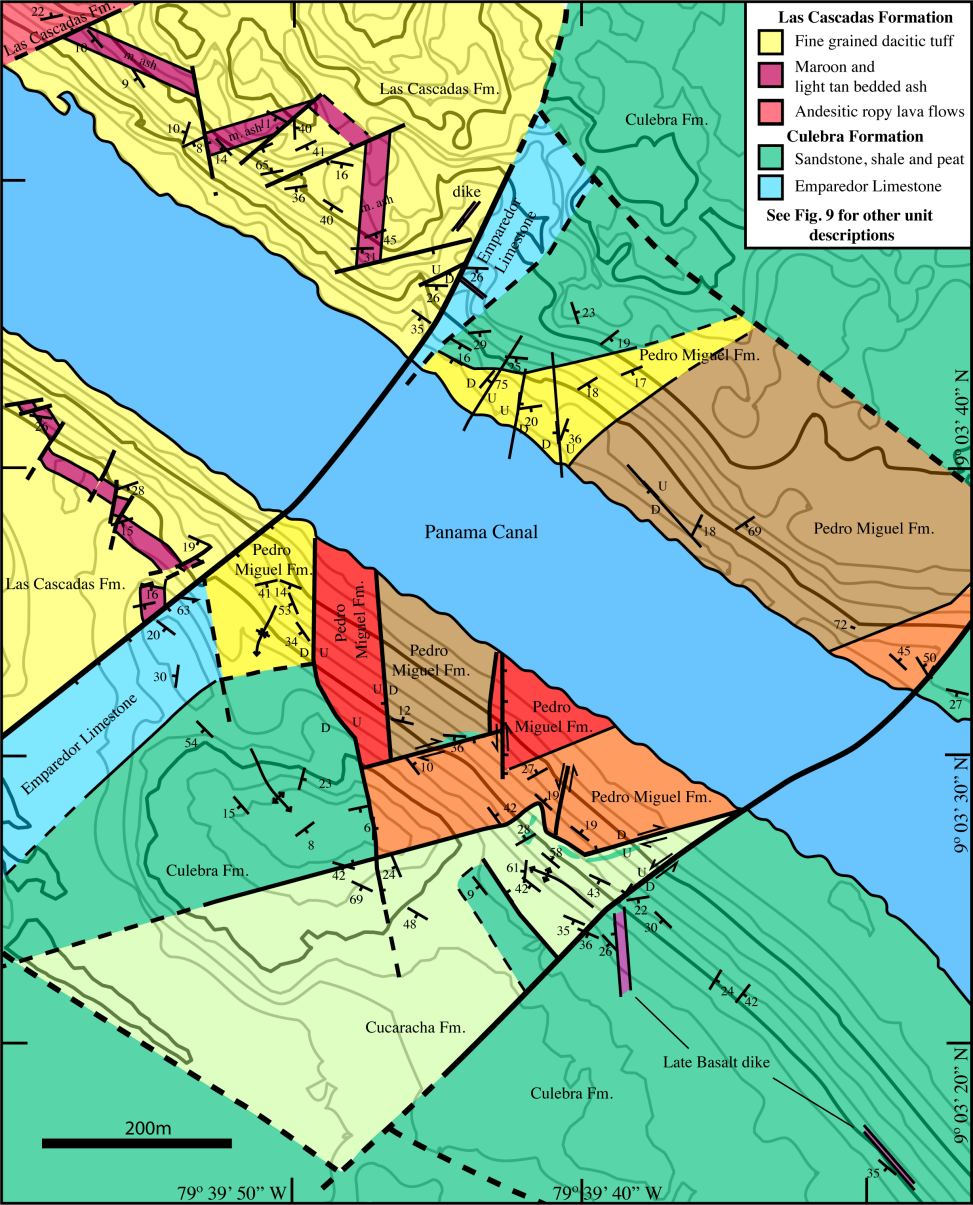
Empire reach geologic map. This map contains the northernmost exposure of the Pedro Miguel Formation along the Panama Canal. At this location, the Pedro Miguel volcanic edifice has been crushed by faulting to a degree that it is not stratigraphically coherent. A large normal fault separates the Pedro Miguel and underlying Cucaracha and Culebra Formations from the Las Cascadas Formation to the northwest. A large left-lateral strike-slip fault also bounds the Pedro Miguel body to the southeast.

Rocks along the Panama Canal have been described and mapped periodically over 100 years since its construction (Fig 1). Geologic surveys were conducted during the initial excavation of the Culebra Cut in the early part of the 20^th^ century (e.g.[28]). Additional geologic mapping was undertaken in the late 1960’s during a previous canal expansion [29] and further regional geologic mapping was conducted by Stewart and Stewart [30]. Each of these earlier efforts had particular geologic or engineering goals on which they focused. For example, the earliest mapping efforts were focused on determining rock type variation in furtherance of Canal construction, whereas Lutton and Banks [29] focused on understanding periodic landslides along the Canal cuts. Much of the recent geologic fieldwork along the Canal has focused on stratigraphy of the sedimentary sections and often has had a paleontologic emphasis (e.g. [31, 32, 33]). The goal of mapping presented here is to improve the understanding of the volcanic stratigraphy and structural geometry along the Panama Canal. Fig 2 presents a stratigraphic section from a volcanic perspective. In contrast, most previous Canal region stratigraphic work has focused almost exclusively on the sedimentary rocks, which make up <25% of the stratigraphic column. Figs 3–8 present field and photomicrographs of the various volcanic units, and Figs 9–13 present geologic maps and cross-sections. This research takes advantage of significant new exposures created by the 2008–2016 Canal expansion, and permission for the fieldwork was granted by the Panama Canal Authority (ACP).

### Description of volcanic units

#### Bas Obispo formation

The Late Oligocene Bas Obispo Formation is the oldest geologic unit that outcrops along the Panama Canal from Panama City to Gamboa (Figs 1 and 2). Stewart and Stewart [30] assigned an Oligocene age based on stratigraphic relationships to fossiliferous strata. However, the Bas Obispo Formation does not contain fossils and is fault bounded making stratigraphic age determinations difficult. Rooney et al. [34] examined rocks from Cerro Patacon that yielded an Ar/Ar age of 25.37 ± 0.13 Ma. The Cerro Patacon rocks are hornblende bearing andesite that sit stratigraphically below the Las Cascadas Formation. Traditionally, rocks at Cerro Patacon have been mapped as part of the poorly defined Panama Formation [30], however compositional, stratigraphic, and temporal similarities to the Bas Obispo Formation indicates geologic continuity.

The Bas Obispo Formation is composed of dark gray to black welded basaltic pyroclastic deposits that contain abundant rounded and vesiculated lava clasts and blocks (Fig 3). Unit thickness is estimated at between 300–1500m, with the large range being due to structural uncertainty and the difficulty in correlating individual outcrops. Individual volcanic beds are not clearly defined, however there is a moderate volcanic clast alignment and variations from clast rich to clast poor layers. Such markers can be used to identify a general westward dip of between 14 to 50 degrees depending on outcrop. The unit is also folded.

Bas Obispo Formation matrix is typically black to dark green in color and contains abundant plagioclase microlites. Large (5–10 mm) hornblende crystals are present in volcanic clasts and small crystals (1–2 mm) are present in the matrix (Fig 3). In thin-section, euhedral plagioclase crystals with minor oscillatory zoning are present, however most plagioclase grains have been broken into angular fragments. Irregularly shaped zones of glass are present in the matrix and exhibit cuspat / lobate margins. Amphibole is much less abundant than plagioclase, and does not appear to be hornblende senso-stricto. It is clear to brown in color with only mild pleochroism and exhibits bight second to third order birefringence colors. It is perhaps paragasitic or an oxyhornblende in composition. Small elliptical to square Fe-Ti oxide minerals are also present. Overall, rocks from the Bas Obispo Formation are dominantly welded basaltic pyroclastic flows with abundant vesiculated lava clasts.

#### Las cascadas formation

The Las Cascadas Formation sits directly above the Bas Obispo Formation, but is fault bounded (Fig 1). It is composed of silicic ash and tuff units interbedded with layers of reddish ropey andesitic lavas (Fig 4). Radiometric ages constrain deposition to between 20.5–25 Ma. This age range comes from the underlying Bas Obispo Formation mentioned above and the age of younger Culebra Formation. Bloch et al. [35] report a U-Pb zircon age of 20.93 ± 0.17 Ma age on an ash layer within the Las Cascadas Formation. In addition, Montes et al., [33] report a U/Pb zircon age on an ash in the lower part of the Culebra Formation of 19.3±0.4 Ma. This suggests that the Las Cascadas Formation was deposited between about 20 and possibly as old as 25 Ma.

As stated above, the Las Cascadas Formation is composed of subarial ash fall tuffs and andesitic lavas. Many of the silicic tuffs have been weathered into yellow to red paleosols. Also present are distinctive black vitreous obsidian layers. In thin section, one of the most distinctive characteristics is the absence of amphibole. Clinopyroxene is present, but not abundant. Silicic tuffs are dominated by devitrified glass and flow structures with occasional euhedral plagioclase crystals. Lapilli ball-like grains that contain abundant small plagioclase crystals are also present. The black obsidian layers are composed dominantly of volcanic glass and pyroxene phenocyrsts that have been highly rounded either by eruptive processes or chemical reabsorption with the surrounding melt. In comparison to the Bas Obispo Formation, Las Cascadas Formation volcanic rocks are significantly more silicic, more highly welded and lack hydrous minerals.

#### Culebra formation

The Miocene Culebra Formation is a marine sedimentary unit composed of organic rich black sandstones and mudstones deposited between 19–20 Ma as discussed above. Early in its stratigraphic sequence there exist limestone and patchy coral reefs that are collectively referred to as the Emperador Limestone. Kirby et al. [31] conducted Sr isotope dating of the limestones, which yielded ages of 23.07 ± 0.53 Ma at the base and 19.12 ± 0.42 at the top. The U-Pb zircon date for the ash within the Las Cascadas Formation suggests that the ca. 23 Ma estimated for the base is biased by reworked material. Thickness estimates for this unit also vary depending on how fault bounded stratigraphic sections are correlated. Kirby et al. [31] suggests a unit thickness of >250 m based on the correlation of sections in the Hodges and Empire sectors of the Canal. Whereas, Montes et al. [33] suggest a minimum thickness of 88 m based on a contiguous section in the Hodges Hill sector. A Culebra Formation thickness of 130 m is used in the Fig 2 stratigraphic section. This thickness is constrained by drill cores from Lutton and Banks [29], but it is a minimum.

#### Cucaracha formation

The Cucaracha Formation is composed of terrestrial tan, reddish and green mudstones and claystones interbedded with fluvial conglomerates and sandstones. Terrestrial animal and plant fossils such as trees are abundant. Previous workers have used such fossil evidence to indicate ages between 19–14 Ma [31]. However, the best age constraints come from a distinctive ash layer known as the Cucaracha Ash, that other workers have used as a marker bed for stratigraphic reconstructions [31].

The Cucaracha Ash itself is a distinctive silicic welded tuff located in the upper portion of the Cucaracha Formation (Fig 5). MacFadden et al. [36] present two overlapping isotopic ages from this layer: An Ar/Ar age of 18.96±0.90 Ma and a U-Pb zircon age of 18.81±0.30 Ma. MacFadden et al. [36] also conducted a magneto-stratigraphy study on the Centenario Bridge section of the Cucaracha Formation and found that it had reversed polarity. This is consistent with the unit being located in the C5Er chron of the geomagnetic polarity time scale and the Cucaracha Formation having an age of approximately 19 Ma.

#### Pedro miguel formation

The Pedro Miguel Formation has a depositional contact with the underlying Cucaracha Formation, and consists of a sequence of basaltic lava flows and subaerially deposited basaltic through andesitic tuffs (Fig 6). Wegner et al. [19] reports an Ar-Ar age of 18.4± 1.07 Ma, but the exact location of samples used to obtain this date is uncertain. The thickness of the unit has significant spatial variability, but is locally greater than 200 m. The thickest portions of the unit occur in locations that exhibit inward dipping bowl-shaped stratigraphy, and are interpreted as individual volcanic breccia pipes or maars. Away from these locations, the unit thins considerably. The Pedro Miguel Formation also has a definite sequence of volcanic sub-units. These sub-units were initially defined based on field mapping and observations at Hodges Hill, but occur in most large exposures. The sub-units also have differing geochemical signatures, but these will be discussed later in the text.

The Hodges Hill map location discussed below contains what we consider to be the “type” volcanic stratigraphy for the Pedro Miguel Formation. The stratigraphy observed there contains the following 5 sub-units from oldest to youngest: 1) Initial silicic layered pyroclastic deposits, 2) Basaltic lava flows (Fig 6A and 6B), 3) Steeply dipping welded tan pyroclastic deposits with well developed bedding planes (Fig 6A and 6C), 4) Massive pyroclastic deposits composed of angular fragments and large remobilized blocks (1 m +), and 5) Shallowly bedded black to gray pyroclastic deposits composed of angular fragments (Fig 6D). These five units are found at Hodges Hill, and are present to a lesser degree at other locations. The most common sequence is the transition from stratigraphically lower layered and highly welded tan pyroclastic deposits to the overlying massive and highly fragmented black pyroclastic deposits.

#### Late basalt formation

The Late Basalt Formation is an informal name for the stratigraphically youngest volcanic unit exposed along the Culebra Cut. Ar/Ar age dating attempts of this unit have so far been unsuccessful, but based on stratigraphic relationships it is conservatively estimated to be older than 15 Ma. The Late Basalt Formation is composed of basaltic to basaltic andesite sills, dikes and flows (Fig 7). The sills and dikes intrude locally into the Culebra, Cucaracha and Pedro Miguel Formations (Fig 7B), and where present, bedded lava flows occur conformably on top of the Pedro Miguel Formation pyroclastic units (Fig 7C). In addition, the Late Basalt Formation occurs over a large area west of the Panama Canal (Fig 1, see Miocene basalt unit). The unit can be differentiated from basaltic lava flows within the Pedro Miguel Formation both in terms of stratigraphic position, structural configuration, and texture / composition. The Late Basalt is most identifiable as larger sills / plugs that are several hundred meters thick and can be up to 500–1000 m in diameter. These sills often have well developed, near vertical columnar jointing in the upper parts of individual intrusions (Fig 7A). However, some bodies contain columnar joints that radiate in multiple directions. They differ from the Pedro Miguel volcanic bodies in that they are composed entirely of magmatic rocks and do not contain pyroclastic units. In terms of petrographic characteristics, the Late Basalt is characterized by strongly aligned plagioclase lathes (Fig 7D). Plagioclase is the dominant phenocryst. Clinopyroxene, where present, occurs primarily as sub- to anhedral interstial crystals located in-between plagioclase grains. This differs from the Pedro Miguel basaltic lava flows in which large euhedral clinopyroxene phenocrysts are present. The Late Basalt Formation is the youngest volcanic unit along the southern Panama Canal.

#### Panama city formation

The Panama City Formation is an informal name for a group of compositionally similar andesitic-dacitic plugs and lava flows located near the Pacific entrance to the Panama Canal (Fig 1). It is the southernmost Canal volcanic unit. Also, this formation is spatially associated with the Late Basalt, but lacks clear stratigraphic relationships with other volcanic units along the Panama Canal. The Panama City Formation has not been dated radiometrically, but intrudes into what is mapped by Stewart and Stewart [30] as the La Boca Formation. The La Boca Formation has been interpreted by Kirby et al. [31] to be the lower part of the Culebra Formation, which has an age of approximately 19–20.5 Ma. However, what has been previously mapped as La Boca Formation near the mouth of the Panama Canal is lithologically different than the rocks Kirby et al. [31] reassigned. The Culebra Formation in the central part of the Culebra Cut is composed of dirty grey marine sandstones with interbeds of limestone, whereas near the Pacific mouth of the Panama Canal, dacitic plugs intrude into bedded tuffs and volcani-clastic sedimentary rocks. Therefore the stratigraphic tie from rocks in the central Culebra Cut to the mouth of the Canal is not particularly strong. However, due to the spatial association and geochemical links (see below) with the Late Basalt, our interpretation is that Panama City Formation rocks are amongst the youngest along the Canal.

The largest volcanic body is the Ancon Hill dacite. It forms the most topographically significant hill in Panama City, and is composed almost entirely of a fine-grained porphyritic dacite / rhyolite. The Ancon Hill dacite contains euhedral phenocrysts of mostly plagioclase with minor potassium feldspar surrounded by a matrix of plagioclase microlites (Fig 8). The microlites are of moderate size and form a quasi-interlocking framework, but still have minor amounts of glass within interstial spaces. Also, present are eudedral to subhedral square phenocyrsts of opaque oxide minerals.

Other Panama City Formation volcanic rocks include a series of andesite/ dacite lava bodies that comprise the three islands at the end of the Amador Causeway, and an andesitic intrusive body near the western abutment of the Bridge of the Americas (Fig 1). The Amador Causeway bodies are intruded by 10+ m wide basaltic-andesite dikes, and well developed columnar andesites are present at this location. The Bridge of the Americas intrusion exhibits sharp near vertical contacts that truncate the host tuff and volcani-clastic sedimentary rocks. This intrusion also contains well-developed magma mingling structures with dacitic liquids locally injected into the dominant andesitic body (Fig 8). Overall, the Panama City Formation bodies are largely intrusive, but observed characteristics suggest a shallow origin.

### Panama canal geologic maps

#### Culebra cut geologic map

The Culebra Cut (also known as the Gaillard Cut) is the part of the Canal that traverses the continental divide and has been subject to the greatest excavations. Fig 9 shows our new geologic map of the Culebra Cut region. This area is dominated by approximately six volcanic complexes that intrude into and are deposited on top of the underlying sedimentary Culebra and Cucaracha Formations. The volcanic complexes can be divided into those dominated by pyroclastic rocks and those dominated by basaltic sills and lava flows. The pyroclastic rocks are classified as part of the Pedro Miguel Formation and the basaltic edifices are part of the Late Basalt Formation as described above. When the Late Basalt is extrusive, it sits conformably above the Pedro Miguel Formation, however in the Culebra Cut, the Late Basalt Formation occurs dominantly as intrusive sills. Hodges and Contractors Hill contain the best exposures of the Pedro Miguel Formation pyroclastic rocks, and each contains a similar sequence of volcanic stratigraphy.

Zion and Gold Hills and Cerro Lirio and Escobar (Fig 9) are comprised of the Late Basalt Formation. Near the contacts, host rock bedding steepens significantly. In map view, the basalt bodies have an elliptical Canal-parallel shape. On Canal parallel sides, host rock bedding contacts are steepened, but are still contact parallel. On Canal perpendicular sides, such as the southeast tip of Cerro Escobar, host rock bedding is at a high angle to and truncated by the basalt contact. Also at Cerro Escobar, Cucaracha Formation rocks are found beneath (NE side) and above the (SW side) basalt body, clearly indicating that the basalt intruded into the older Cucaracha Formation (Fig 10).

In terms of internal structure, the basalt bodies are fine grained, but exhibit observable magmatic fabrics and compositional variation. In terms of mineralogy, the bodies are dominated by plagioclase, but vary in the amount of pyroxene phenocrysts that are present. Pyroxene is more common in the lower parts of each of the bodies, and in Gold Hill and Cerro Lirio this variation has been mapped as a separate unit. Also present is cryptic centimeter-scale compositional layering with each layer having variable amounts of plagioclase and clinopyroxene. The small-scale compositional bands align with uniformly oriented plagioclase lathes that can be clearly observed in thin-section (Fig 7). This alignment of plagioclase lathes is what defines the magmatic fabric. Overall, the compositional bands tend to be aligned with adjacent basalt body contacts.

Based on mapping presented here and Lutton and Banks [29] drill core data, cross-sections across the Culebra Cut have been drafted (Fig 10). The drill core data provide clear constraints as to the geometry of the Late Basalt bodies. Drill cores penetrate the Zion Hill and Cerro Lirio basalt bodies and enter the underlying Cucaracha Formation sediments at elevations of 25 m above mean sea level (msl). This indicates they have flat bottoms, and are therefore large sills with a thickness of approximately 200m.

Gold Hill has a different type of geometry. Its upper portion is composed of Late Basalt with cryptic plagioclase layering, but the basalt body sits on and is surrounded by a corona of steeply dipping bedded pyroclastic deposits. Drill cores through the center of Gold Hill extend to depths of -200 m below sea level and show that the marginal pyroclastic deposits extend underneath the basalt bodies and form a bowl-like structure. Previous mapping [29] also identified a basalt flow at the base of the pyroclastic deposits, however subsequent canal widening has removed that outcrop. Basalt flows in a similar stratigraphic position at Hodges Hill are in the Pedro Miguel Formation (see below). In total, the thickness of volcanic rocks at Gold Hill is over 350 m. Overall, the structure has a cylindrical geometry composed of 2/3 pyroclastic rocks and 1/3 basalt.

In addition to sedimentary and volcanic events, the Culebra Cut also experienced significant fault activity, and is cross-cut by an orthorhombic fault set. The faults cut both sedimentary and volcanic rocks, although the larger volcanic edifices appear to deflect the faults somewhat. The faults are normal and left-lateral strike-slip in nature. Normal faults are in general cross-cut by strike-slip faults, however almost all of the faults have displacements of <100m. The largest fault is a Canal parallel normal fault that sits just to the NE of Gold Hill. Drill cores (Fig 10) constrain the offset on this fault to approximately 100 m. This fault dips to the SW, but other normal faults dip to the NE, and collectively these form a small Canal parallel graben system that is illustrated in the Fig 10 cross-sections.

#### Hodges hill geologic map

Hodges Hill contains the best-exposed sequence of Pedro Miguel Formation volcanic strata. This location preserves a sequence of inward dipping basaltic lava flows and basaltic to andesitic pyroclastic deposits (Fig 11). The volcanic rocks are deposited conformably atop the terrestrial Cucaracha Formation sediments. The first volcanic layer at this location is an andesitic welded tuff deposited within the Cucaracha Formation (e.g. the Cucaracha Formation Ash). The layer is approximately 5 m thick, and it has been used as a marker bed to stratigraphically tie geographically separated parts of the Cucaracha Formation together. The contact of the Hodges Hill volcanic edifice is depositional at its margins, but locally small late normal faults with up to 1 m of displacement are present. We interpret these small discontinuous faults as being related to the subsequent construction of the volcanic edifice on soft shales and paleosols.

The first layer in the contiguous volcanic edifice is an andesitic welded tuff with graded beds 0.1–1.0 meters thick. There are variations in the degree of welding that give the layers a black to white banded appearance. The next mapped layer consists of a sequence of basalt flows 40–50 m thick. This sequence is composed of basalt flows with vesiculated tops and individual flow thicknesses of 1–5 m. The flows contain euhedral plagioclase and pyroxene phenocrysts (Fig 6), and do not exhibit the strongly layered textures observed in the Late Basalt Formation sills. Locally, there are volcaniclastic interbeds between the lava flows.

On the southern part of the complex there is a sequence of bedded andesitic maroon ash and tuff. It is not welded, and locally the tuff body has volcaniclastic channel deposits. On the northern side of the edifice, the maroon ash is absent.

Deposited on top of the basalt flows in the north, and the ash in the south, are bedded basaltic pyroclastic deposits. These are welded and have highly linear beds that vary from 10–50 cm thick. In thin-section, glass stringers are deflected around pyroclastic lithic fragments (Fig 6). Locally, mm to cm-scale pyroclastic fragments are embedded in a white quartz matrix. The quartz matrix likely formed due to percolation of syn- to post depositional fluids with significant dissolved silica. Bedding dip is relatively steep at 30–50°. In fresh to slightly weathered outcrops this unit has a distinctive tan color. Finally, the tan bedded pyroclastic map unit is a diagnostic component of the Pedro Miguel Formation.

On the north side of Hodges Hill, the next map unit is a moderately fine-grained black pyroclastic deposit that contains basaltic bombs, and along internal contacts, fragments of carbonized wood. There is an angular unconformity between the steeply dipping welded tan pyroclastic deposits and more shallowly bedded black pyroclastic unit discussed in this paragraph.

On the south side of Hodges Hill, a highly brecciated unit that contains large, variously rotated, tan pyroclastic blocks sits above the contiguous bedded tan pyroclastic map unit. Blocks of the tan bedded unit range in size from cm-to-10’s of meters across, and they sit in a matrix of the black pyroclastic material. The fragmentation of the tan bedded pyroclastic unit also extends to the sub-mm scale and similar fragmentation is observed in thin-section (Fig 6).

The final volcanic unit at Hodges Hill is a somewhat coarser grained version of the black pyroclastic unit that exhibits progressively more shallow bedding dips towards the top of Hodges Hill. This unit is bedded and has visible cm-scale pyroclastic fragments. Pedro Miguel Formation volcanic rocks continue to the southwest, but cannot be mapped in detail due to weathering and tropical forest cover.

In addition to the mapping, two cross-sections have been constructed through Hodges Hill to illustrate its internal structure. The cross-section perpendicular to the Panama Canal is constrained further with drill-core data from Lutton and Banks [29]. These cross-sections illustrate the inward dipping and near symmetrical volcanic structure exhibited at Hodges Hill.

Drill core data indicate that the highly brecciated pyroclastic units extend at least 150m downward and potentially further as the central drill core ends within the volcanic units.

Our interpretation is that the highly brecciated unit formed during an explosive eruption that remobilized the earlier bedded tan pyroclastic unit and blasted out the central portion of Hodges Hill. The uppermost black shallowly dipping units were subsequently deposited on top of the now filled central region. Overall, the volcanic activity at Hodges Hill can be organized into two groups, each of which initiated with an eruption of silicic material. The first was largely effusive with basaltic lava flows and pyroclastic activity gentle enough to create well-bedded deposits. However, enough volcanic explosivity must have existed to create the observed bowl like structure, which must have been present at this time due to observed angular bedding discordances between basalt flows and tan pyroclastic deposits. The second volcanic episode initiated with the maroon andesitic tuff, and is characterized by explosive volcanism. The eruptive force in this episode must have been great enough to partially destroy the previous volcanic edifice and to mobilize blocks 10’s of meters across. Products from the explosive phase completely filled the inward dipping Hodge’s Hill structure to produce what we observe today.

#### Cartagena hill geologic map

In the Culebra Cut region, faults are deflected around the volcanic bodies. However at Cartagena Hill (Fig 12), a Pedro Miguel Formation edifice has partially been dismembered by subsequent fault activity. The volcanic stratigraphy at this location is similar, but simplified compared to Hodges Hill. The lowest members of the Pedro Miguel Formation are bedded, welded tan pyroclastic rocks. This unit has bedding thicknesses that range from 0.1–1.0 m, and are moderate coarse grained. Also, the bedded pyroclastic unit was deposited conformably on top of Cucaracha Formation sedimentary rocks. Above the bedded pyroclastic unit sits a thick (100 m +) sequence of massive pyroclastic breccia that contains meter-sized blocks of the underlying unit. The overall geometry of the Cartagena Hill edifice is an inward dipping bowl similar to what is described at Hodges Hill. However, normal faults have dismembered its original geometry and exposed a horst of Cucaracha Formation sediments within Cartagena Hill.

At the time of our mapping (summer 2010), the upper 100 m of Cartagena Hill had been excavated as part of the Panama Canal expansion process. The channel from the new locks enters the original Panama Canal at this location and so the remainder of Cartagena Hill has subsequently been removed. Stewart and Stewart [30] show the unexcavated geology, and these observations have been incorporated into the Fig 12 cross-section. These indicate that a body of basalt existed at the top of Cartagena Hill. Our interpretation is that these were part of the Late Basalt because at a nearby hill, vesiculated Late basalt flows are observed conformably on top of massive Pedro Miguel Formation pyroclastic rocks (Fig 7C). At the mapping elevation (60 m msl), several basaltic dikes, up to 20 m thick, are exposed in the Cucaracha Formation horst. These dikes are cut by normal faults that truncate the entire complex, but our interpretation is that these were feeder dikes for the now excavated Late Basalt body shown in Stewart and Stewart (1980). Overall, Cartagena Hill is a Pedro Miguel Formation bowl shaped edifice that exhibits a two-phase (first bedded, second brecciated pyroclastic rocks) eruptive sequence similar to Hodges Hill, however Cartagena Hill has been partially dismembered via normal faults.

#### Empire reach / Lirio Norte geologic map

The northernmost exposure of the Pedro Miguel Formation occurs at Empire Reach (also known as Lirio Norte) along the Panama Canal (Fig 13). This location contains Pedro Miguel Formation sub-units (e.g tan bedded-welded pyroclastic rocks) that are recognizable from other areas; however, they are not configured as a coherent volcanic edifice. We have divided Pedro Miguel Formation rocks into four sub-units at this location. These include: tan bedded and welded pyroclastic rocks, massive brecciated pyroclastic rocks, massive basalt and black to white bedded tuffs. Also at this location, each Pedro Miguel sub-unit is largely fault bounded. On the south side of the Canal, the Pedro Miguel complex forms a wedge-shaded body in which the various sub-units have a fault bounded orthorhombic geometry. In addition, there are differences between Pedro Miguel Formation rocks exposed on each Canal bank. On the north side of the Canal, Pedro Miguel rocks are more stratigraphically continuous, but have less lithologic variability. The initial black and white tuffs have a bedded boundary with a large section of massive pyroclastic breccia. Finally, bedded tan pyroclastic rocks on the south side of the complex are continuous between Canal banks. Overall, the Pedro Miguel Empire Reach exposure exhibits more structural complexity and less stratigraphic coherence than other localities despite almost 100% exposure due to recent excavations.

In addition to internal faulting, the entire Empire Reach Pedro Miguel complex is bounded between two large faults. The southern fault has left-lateral strike-slip kinematics, whereas the northern fault is normal in nature. North of the large normal fault, the Las Cascadas Formation is cut by a series of smaller NE-SW trending normal faults that produce a series of 50–100 m scale horst-and-graben structures.

The sedimentary rocks underlying the Pedro Miguel Formation at Empire Reach have also been faulted and folded. Immediately south of the large northern normal fault, are exposures of the Emperador Limestone, which Kirby et al. [31] placed as a member in the lower part of the Culebra Formation. On the north side of the Canal, the Emperador Limestone contains coral heads up to 1 m across. The Emperador limestone grades into marine Culebra Formation sandstones, and in locations nearest the Canal, into black and white Pedro Miguel Formation tuffs. The initial black and white tuffs are interbedded with marine Culebra Formation sandstones at this location. On the south side of the Canal, the black and white tuffs and marine sandstones exhibit 25–100 m scale folds that abut against the northern side of the Pedro Miguel Formation body. On the south side of the Pedro Miguel body, Cucaracha Formation paleosols and clays are faulted against the Pedro Miguel rocks (Fig 13). Fault formed windows within this wedge of Cucaracha Formation expose the underlying Culebra Formation sandstone.

The existence of Cucaracha Formation rocks to the south and Culebra-Formation-like rocks to the north suggest a paleo-environmental change from terrestrial (south) to marine (north) during eruption of Pedro Miguel Formation rocks at this location. Traditionally, the marine Culebra Formation proper lies underneath the terrestrial Cucaracha Formation (e.g. [33]), but what we observe surrounding the Empire Reach complex suggests a lateral depositional environmental change at the time of Cucaracha Formation deposition. Finally, the structural complexity at Empire Reach is attributed to the two large faults between which the Pedro Miguel Formation complex is sandwiched.

## Geochemistry of canal zone rocks

### Methods

**Fig 14:**
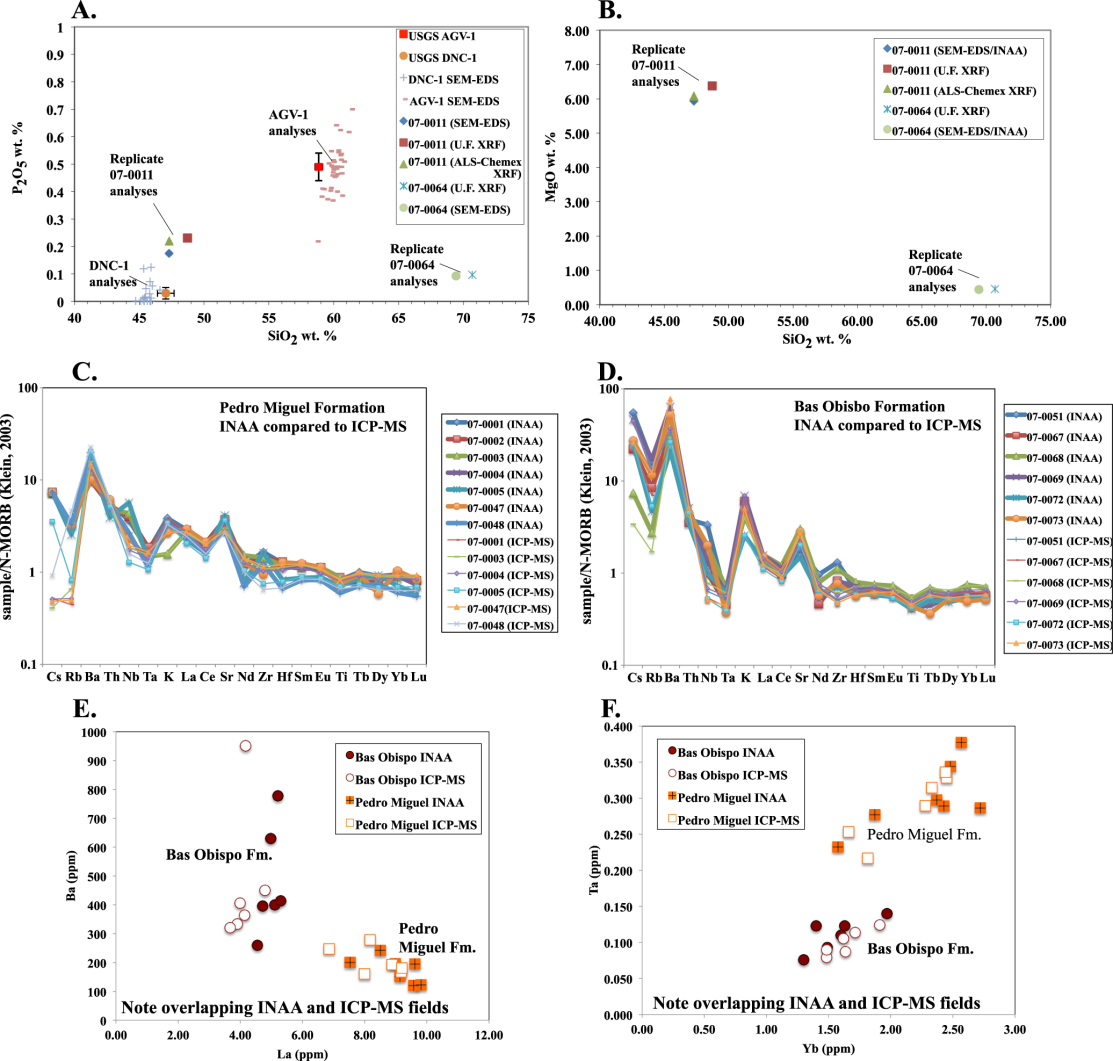
Comparison of major and trace element chemistry to standards and replicate analyses using multiple labs and techniques. A) P_2_O_5_ vs. SiO_2_ wt. % standard and replicate analyses, B) MgO vs SiO_2_ wt. % replicate analyses, C) Replicate analysis of Pedro Miguel Fm. rocks using INAA and ICP-MS, D) Replicate analysis of Bas Obispo Fm. rocks using INAA and ICP-MS, E) Ba vs. La plot of INAA and ICP-MS analyses from the Pedro Miguel and Bas Obispo Fm.’s, F) Ta vs. Yb plot of INAA and ICP-MS analyses from the Pedro Miguel and Bas Obispo Fm.’s,.

Geochemical data presented in this paper are derived from instrumental neutron activation analysis (INAA), x-ray florescence spectrometry (XRF), and scanning electronic microscope energy dispersive spectrometry (SEM-EDS) (S1 Table). All of the trace element data except Nb and Zr are from the INAA technique. Nb and Zr were determined using XRF. The presented major element data are also derived from INAA, except for SiO_2_ and P_2_O_5_, which were determined by low-vacuum SEM-EDS. INAA was conducted at the University of Missouri Research Reactor, whereas the XRF and SEM-EDS analyses were conducted at the Smithsonian Museum Conservation Institute in Washington D.C.. The SEM-EDS analyses were compared to USGS standards AGV-1 and DNC-1, and for example, SiO_2_ wt. % had an accuracy of ± 1.45 wt. % and a precision of 0.45 wt. %. Several samples, using the same powders, were re-analyzed via whole rock XRF techniques at the University of Florida and commercially at ALS-Chemex, using a lithium metaborate flux to create glass disks, and yielded concentrations within the stated accuracy (Fig 14A and 14B). The difference between the reanalyzed XRF samples and the INAA + SEM-EDS analyses was for most elements less than 5%. Overall, duplicate analyses at different laboratories and by analytical techniques yielded very similar elemental concentrations. All analytical techniques were conducted on powdered whole rock samples. To reduce contamination powdering was done using an agate mill. Also, care was taken to sample the freshest available rocks in the field, and only samples that contained unaltered igneous textures in thin-section were chosen for geochemical analysis (e.g. Figs 3–8). The totals presented are anhydrous and do not include LOI. See supporting online material in Farris et al. [16] for a more compete description of the analytical methods.

In addition, for select samples, the INAA trace element data was compared via re-analysis to ICP-MS measurements also using the same powders (Fig 14C–14F). The re-analysis was done using an ELEMENT 2 at the University of Florida. For most trace elements, both INAA and ICP-MS analyses yielded similar concentrations and have elemental values within 5% of each other. However, in the Pedro Miguel Formation Cs and Rb measurements were systematically high in the INAA data set, as actual concentrations appear to be below the limit of analytical precision (Fig 14C). Nb and Zr measurements were also somewhat higher in the INAA / XRF dataset as compared to ICP-MS. However, for most elements, the ICP-MS and INAA data sets plot in overlapping field and agree with one another down to sub-ppm levels of precision. This can be clearly observed in plots of Ba vs La (Fig 14E) and Ta vs Yb (Fig 14F). These elements were chosen as examples because they illustrate some of the key geochemical differences between Oligocene (Bas Obispo Fm.) and Miocene (Pedro Miguel Fm.) units. Note the Ba enrichment and Ta depletion in the Bas Obispo Fm. Rocks relative to the Pedro Miguel Fm. Overall, the dominant trace element patterns are very similar in both the INAA and ICP-MS data sets. The chemistry and figures presented below use the INAA/XRF/SEM-EDS dataset as only they cover all units.

### Major element chemistry

**Fig 15:**
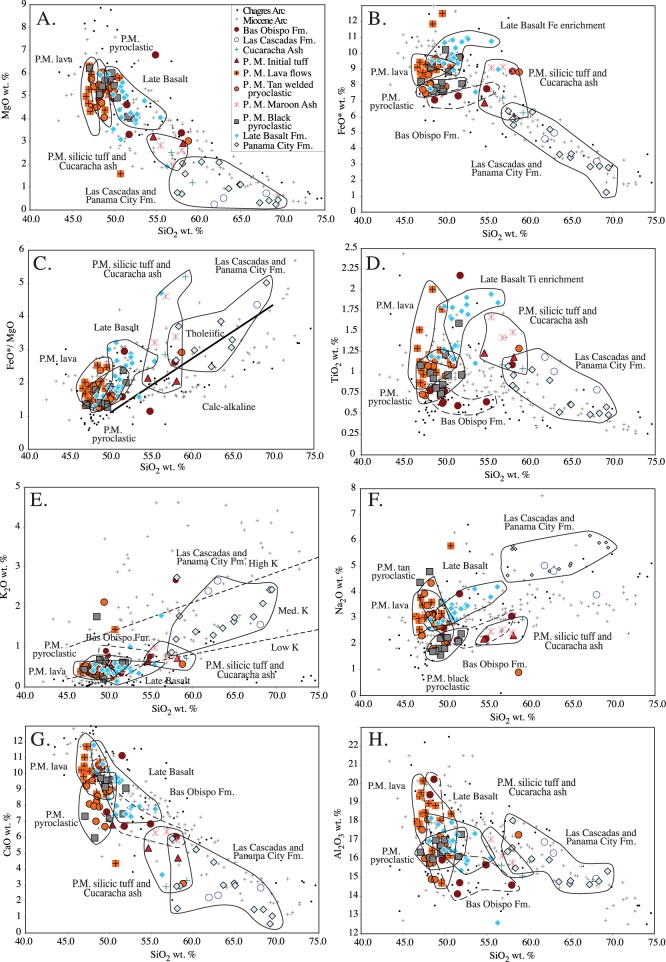
Major element chemistry of the Canal volcanic rocks. The Wegner et al. [19] Chagres and Miocene arc data are plotted for comparison. A) MgO vs SiO_2_ wt. % B) FeO* vs SiO_2_ wt. % C) FeO*/MgO vs SiO_2_ wt. % D) TiO_2_ vs SiO_2_ wt. % E) K_2_O vs SiO_2_ wt. % F) Na_2_O vs SiO_2_ wt. % G) CaO vs SiO_2_ wt. % H) Al_2_O_3_ vs SiO_2_ wt. %.

Major element chemistry of Canal volcanic rocks range from basalt to dacite in composition and from 47 wt. % to 70 wt. % SiO_2_ (Fig 15). The maximum observed MgO is approximately 6 wt. %, and this is found in the Pedro Miguel Formation lava flows (Fig 15A). Within the Pedro Miguel Formation and the Late Basalt, each progressively younger unit exhibits a progressively lower slope MgO and SiO_2_ trend with the Pedro Miguel lava flows being the steepest and the Late Basalt having the lowest slope trend. One exception are Pedro Miguel silicic tuff units which plot at the head of the Late Basalt trend. These rocks are also the most silicic within the Pedro Miguel and Late Basalt units. The most silicic units along the Panama Canal are the Panama City and Las Cascadas Formations, which plot together and range from 57–70 wt. % SiO_2_ and have values of < 2 wt. % MgO.

Overall, Panama Canal volcanic rocks are tholeiitic and even the most silicic rocks are tholeiitic in terms of their FeO*/MgO ratio (Fig 15 C). Another characteristic of tholeiitic rocks is a strong Fe and Ti enrichment due to the early crystallization of plagioclase [37]. Such a trend is observed within the Late Basalt and to a lesser degree in the Pedro Miguel Formation. In terms of FeO* (all Fe as FeO), the Late Basalt Formation increases from 9 to 11 wt. % with increasing SiO_2_ (Fig 15B). In terms of TiO_2_, the Late Basalt Formation increases from 1 to almost 2 wt. % with increasing SiO_2_ exhibiting an even stronger enrichment trend (Fig 14D). For both Fe and Ti, the silicic Pedro Miguel tuffs connect the enriched Late Basalt rocks in a trend continuous with the more silicic Panama City Formation. In contrast, the Bas Obispo Formation does not exhibit similar Fe and Ti enrichment.

The Canal volcanic rocks also exhibit distinctive trends in terms of the alkali elements. The majority of Canal volcanic rocks are low to medium K (Fig 15E). However, the Las Cascadas and Panama City Formation rocks plot in the medium to high K series. In terms of Na_2_O several distinct series are present. At silica values between 47–50% SiO_2_, Na_2_O decreases steeply from approximately 5 to 2 wt. %. At those silica values, Na_2_O is effective at discriminating the different Pedro Miguel pyroclastic units, with the black fragmented unit ploting with the lowest Na_2_O values. At SiO_2_ values greater than 50 wt. %, Na_2_O increases. This is particularly evident in the Late Basalt and the Panama City and Las Cascadas units which together form a continuous trend of increasing Na_2_O with increasing SiO_2_ with 6% Na2O reached at 70% SiO_2_. CaO decreases continuously with SiO_2_ from approximately 12 wt. % CaO at 47 wt. % SiO_2_ to 0.5 wt. % CaO at 70 wt. % SiO_2_ (Fig 15G). This trend is smooth with several exceptions. The tan Pedro Miguel pyroclastic unit extends to lower CaO values, and the Las Cascadas / Panama City group does as well, although at higher SiO_2_ values. Overall, the Canal volcanic rocks have in general low K_2_O values, relatively high Na_2_O and average CaO concentrations for arc volcanic rocks.

### Trace element chemistry

#### Spider plots

**Fig 16:**
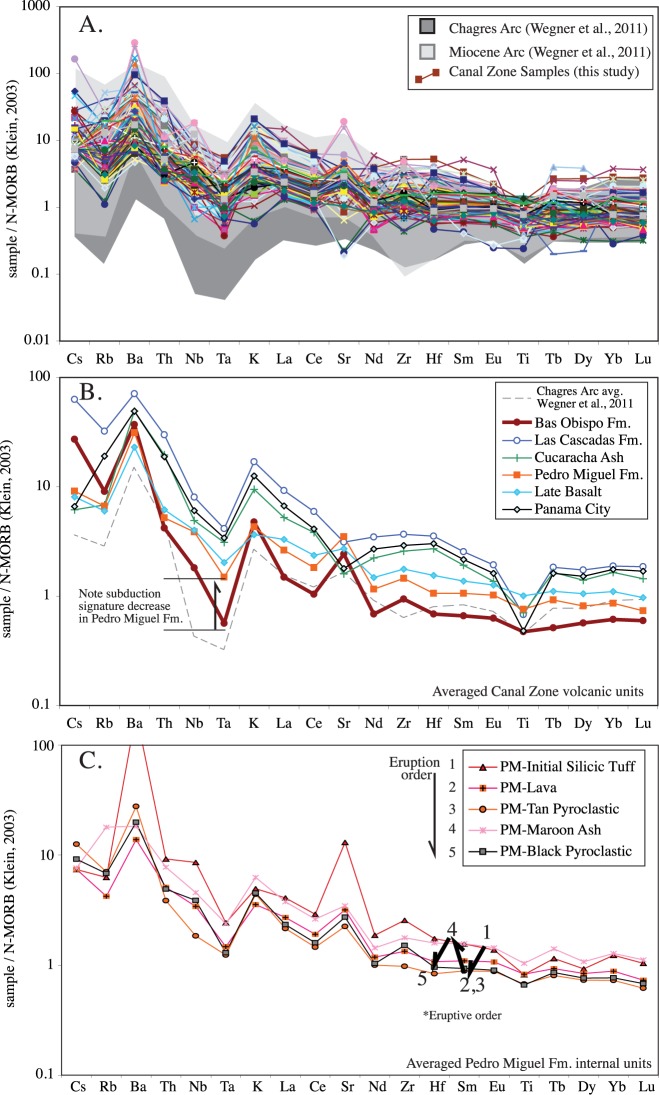
Trace element chemistry of the Canal volcanic rocks presented via MORB normalized spider diagrams. A) All Canal volcanic rock analyses. Wegner et al. [19] data also shown for comparison. The Canal rocks fall roughly within the Miocene arc field. B) Trace element data averaged by unit. Note that the units separated into three main groups: Bas Obispo, Pedro Miguel/Late Basalt, and Las Cascadas/Panama City Formations. C) Pedro Miguel Formation data averaged by sub-unit. Note oscillation between high and low trace element concentrations with unit eruptive order.

In general, the trace element signature of the Canal region volcanic rocks plot within the Miocene arc field as defined by Wegner et al. [19] (Fig 16A), however in detail there are important differences. In Fig 16B, the trace element data has been averaged by unit to identify the important trends between the major Canal volcanic units. The units split into three main groups. The Bas Obispo Formation has significantly lower rare-earth elements (REE), by far the largest negative Ta anomaly, and is relatively enriched in the large-ion lithophile elements (LILE) for a basaltic unit. The Bas Obispo Formation also exhibits large positive Sr and K anomalies and essentially no Ti anomaly. The Pedro Miguel and Late Basalt Formations exhibit intermediate REE concentrations, small Ta anomalies, a moderate positive Sr anomaly and low LILE concentrations. The Late Basalt exhibits consistently higher REE values than the Pedro Miguel Formation, but has somewhat lower LILE’s. The third unit grouping consists of the Las Cascadas Formation, the Cucaracha ash layer and the Panama City Formation. These units are all silicic and have the highest trace element concentrations. Diagnostic trace element features of this group include: A large negative Ti anomaly, a concave down high-field strength element (HFSE) pattern, a negative Sr anomaly, a positive K anomaly and a mix of high and low LILE’s. Overall, each of the three unit groups exhibits a distinct trace element signature. Via geochemical modeling we will address differences in origin or petrologic process that may have given rise to these differences.

In addition, our sampling is detailed enough to examine the Pedro Miguel sub-units for distinctive trends. Fig 16C is a spider plot averaged by the various Pedro Miguel sub-units identified via mapping described above. The Pedro Miguel sub-units in general exhibit relatively tight parallel trends. One exception to this is the high positive Sr and Ba anomalies present in the initial silicic tuff sub-unit. The most striking observation is the oscillation between high and low trace element concentrations throughout the eruptive sequence. Each “cycle” begins with a higher silica, high trace element eruptive event and then transitions to a larger low silica and low-trace element concentration eruptive sub-unit. This sequence occurs twice in the Hodges Hill type locality for the Pedro Miguel Formation.

#### Tectonic discrimination diagrams

**Fig 17:**
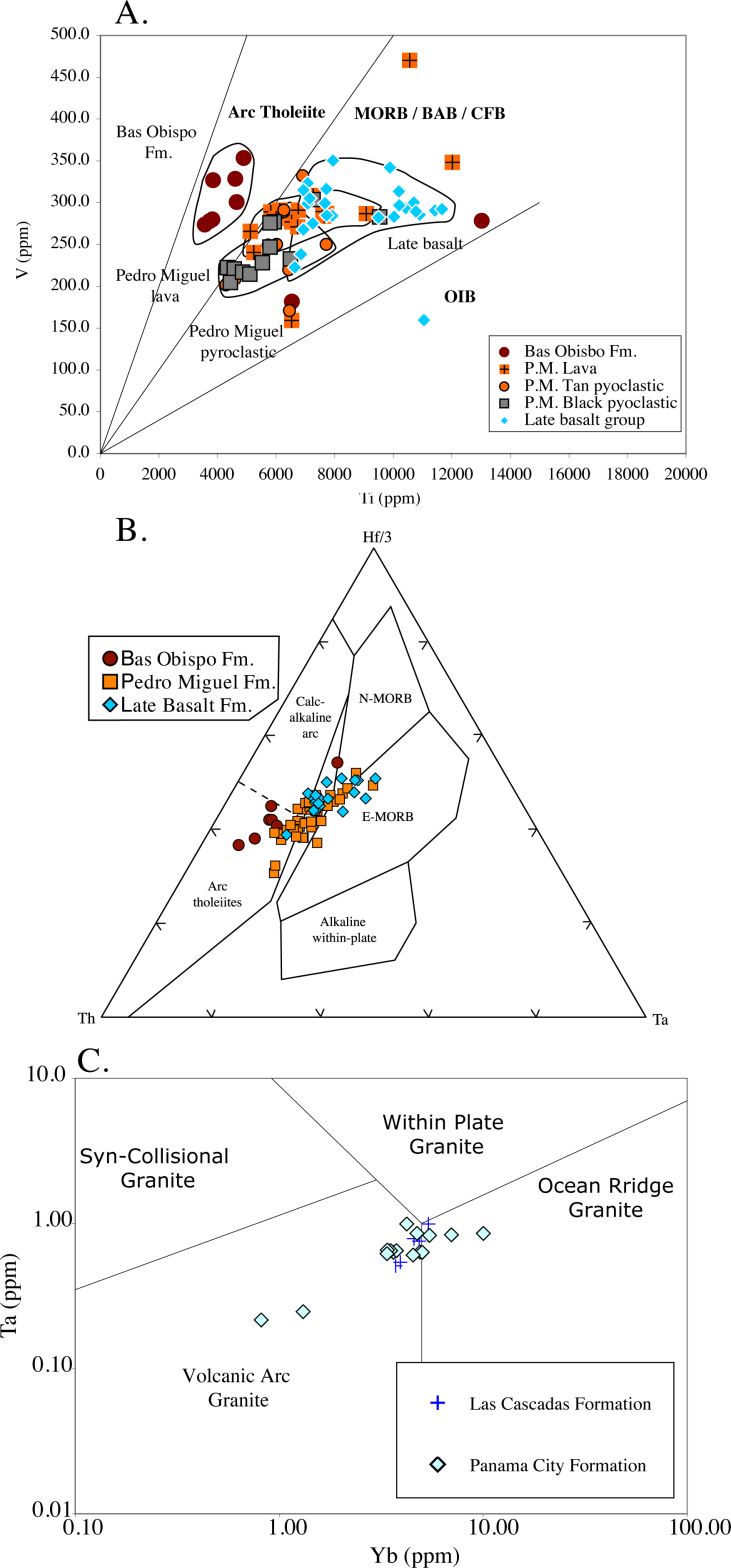
Tectonic discrimination diagrams. A) V vs. Ti diagram of Shervais [38]. This plot sharply divides the Bas Obispo and Pedro Miguel Formations into arc tholeiite and back arc/MORB fields, respectively. B) Hf/Th/Ta diagram of Wood [39]. Pedro Miguel Formation rocks show a continuous trend from arc tholeiite to MORB fields. C) Ta vs Yb diagram of Pearce et al. [40]. This diagram is for rocks of granitic composition. Note Panama City and Las Cascadas Formation rocks trend from the volcanic arc to the ocean ridge granite fields.

Tectonic discrimination diagrams are useful for denoting changing geologic origins of magmatic rocks. For Canal volcanic units, Oligocene rocks plot in arc tholeiite fields, whereas Miocene rocks transition into a mid-ocean ridge / back-arc basin field (MORB/BAB, respectively). This transition is shown most sharply on the V vs. Ti diagram of Shervais [38] (Fig 17A). Here rocks from the Oligocene Bas Obispo Formation clearly plot within the arc tholeiite field, whereas Miocene Pedro Miguel and Late Basalt Formation rocks plot within the MORB/BAB field. On other diagrams, the transition between different tectonic fields is more gradual. On the Hf/Ta/Th diagram of Wood [39], the Bas Obispo Formation rocks once again plot within the arc tholeiite field, but Pedro Miguel and Late Basalt rocks form a continuous grouping that extends from the arc tholeiite field into MORB fields and also straddles the boundary between normal and enriched-MORB (Fig 17B). Finally, we have plotted the applicable Canal volcanic rocks on the Ta/Yb diagram of Pearce et al. [40]. This diagram is designed for more silicic rocks, whereas the two above are primarily intended for basaltic rocks. On the Ta/Yb diagram, the Las Cascadas and Panama City Formation rocks extend from the volcanic arc into the ocean-ridge granite field (Fig 17C). Overall, tectonic discrimination diagrams for both basaltic and silicic rocks indicate a transition to an extensional environment across the Oligocene and Miocene boundary.

#### Trace element ratio plots

**Fig 18:**
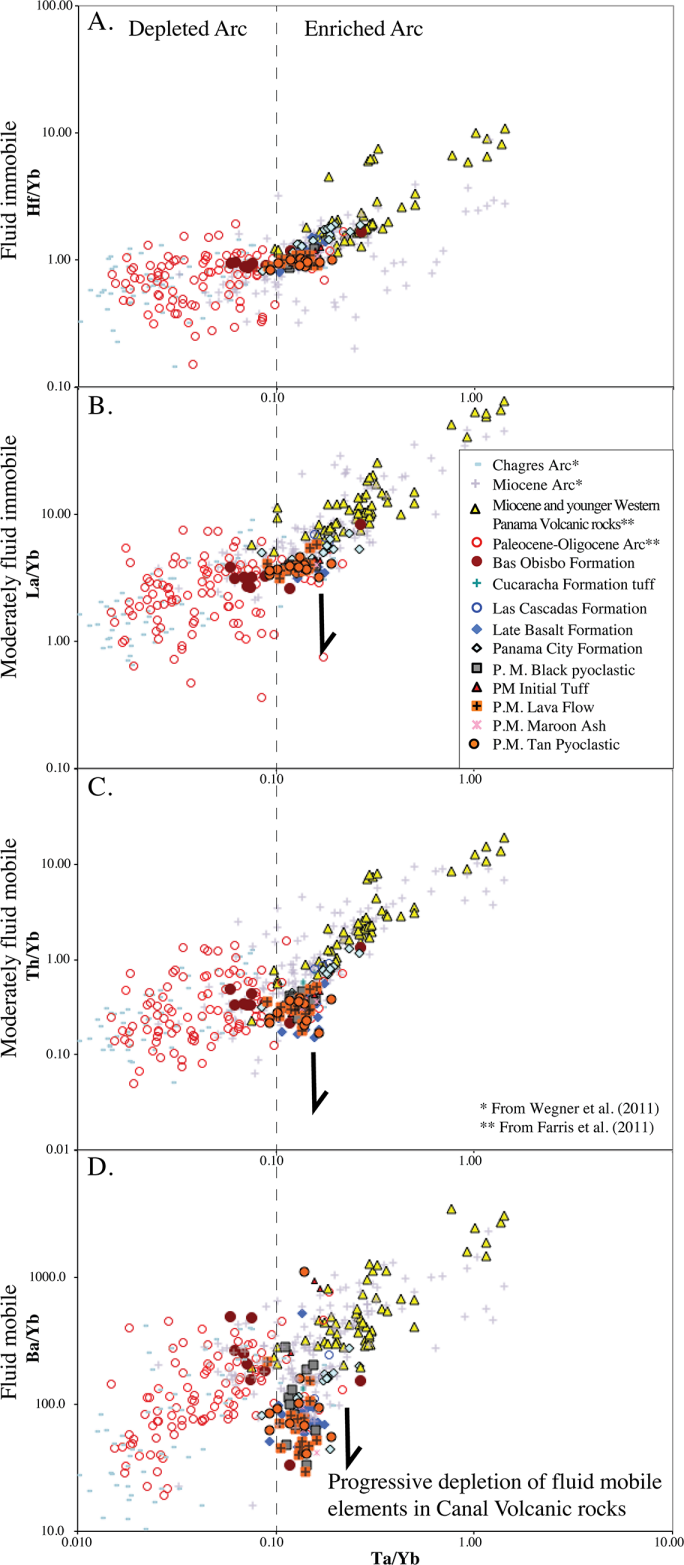
Trace element ratio plots. Farris et al. [16] data from Paleocene-Oligocene arc and Miocene and younger arc are plotted in addition to the Canal region analyses (all are INAA data). Wegner et al. [19] ICP-MS data is also shown for comparison. Note the Canal volcanic rocks are located at a transition between the early depleted arc and the younger enriched arc. Also, Canal volcanic rocks show a distinct negative anomaly in fluid mobile elements. A) Hf/Yb vs. Ta/Yb. B) La/Yb vs. Ta/Yb. C) Th/Yb vs. Ta/Yb. D) Ba/Yb vs. Ta/Yb.

Trace element ratios are tracers of volcanic and mantle processes that allow one to look through upper crustal fractional crystallization [41]. Fig 18 shows Canal volcanic rocks normalized with respect to Yb. This plot shows Hf/Yb, La/Yb, Th/Yb and Ba/Yb ratios with respect to Ta/Yb. The Hf/Yb through Ba/Yb sequence are plotted with respect to increasing fluid mobility with Hf being essentially fluid immobile and Ba being fluid mobile. Also, Canal volcanic rocks of this study are plotted together with older and younger Panama arc rocks from Farris et al. [16] and Wegner et al. [19]. The Farris et al. [16] analyses are INAA data from the same facility as the Canal rocks presented here, whereas the Wegner et al. [19] are ICP-MS data.

These plots show two main trends. First, Wegner et al. [19] divide the Panama arc into a Late Cretaceous-Paleocene Chagres arc derived from a depleted mantle and an enriched Miocene arc. This division is observed on Fig 18 using the Ta/Yb ratio as a discriminant with rocks having a ratio less then 0.1 belonging to the older depleted arc and rocks having a Ta/Yb ratio greater than 0.1 belonging to the enriched younger arc. The Canal volcanic rocks lie at the boundary between these two arc regimes and straddle it with the Oligocene Bas Obispo Formation solidly within the older arc field and the Miocene Canal volcanic rocks within the younger. In addition, with respect to La/Yb and Hf/Yb ratios, the Canal volcanic rocks lie at a geochemical inflection point between trends defined by older depleted and younger enriched arc rocks [16].

The second main observation is the Canal rocks are depleted in fluid mobile elements. A small, but significant, depletion is observed in the moderately fluid mobile Th/Yb ratio. In the strongly fluid mobile Ba/Yb ratio a larger negative anomaly is observed. Overall, the degree of element depletion in Canal volcanic rocks is correlated with the degree of element fluid mobility, and the fluid element depletion itself is a unique characteristic of the Canal volcanic rocks with respect to the older and younger arc throughout Panama.

### Geochemical models

To better interpret the petrologic evolution of the above Canal volcanic rocks, major and trace element geochemical models have been constructed. The models indicate that shallow level fractional crystallization can explain most of the compositional variation within the Pedro Miguel and Late Basalt Formation. However, fractional crystallization alone does not well describe geochemical variation between the Oligocene Bas Obispo and the various Miocene units. Within the Miocene rocks, pure fractional crystallization models have difficulty replicating the most silicic units (e.g. the Las Cascadas Formation) at reasonable melt fractions, however assimilation fractional crystallization models are more successful. Below the details of the major and trace element models are discussed in depth.

#### Major element models

**Fig 19:**
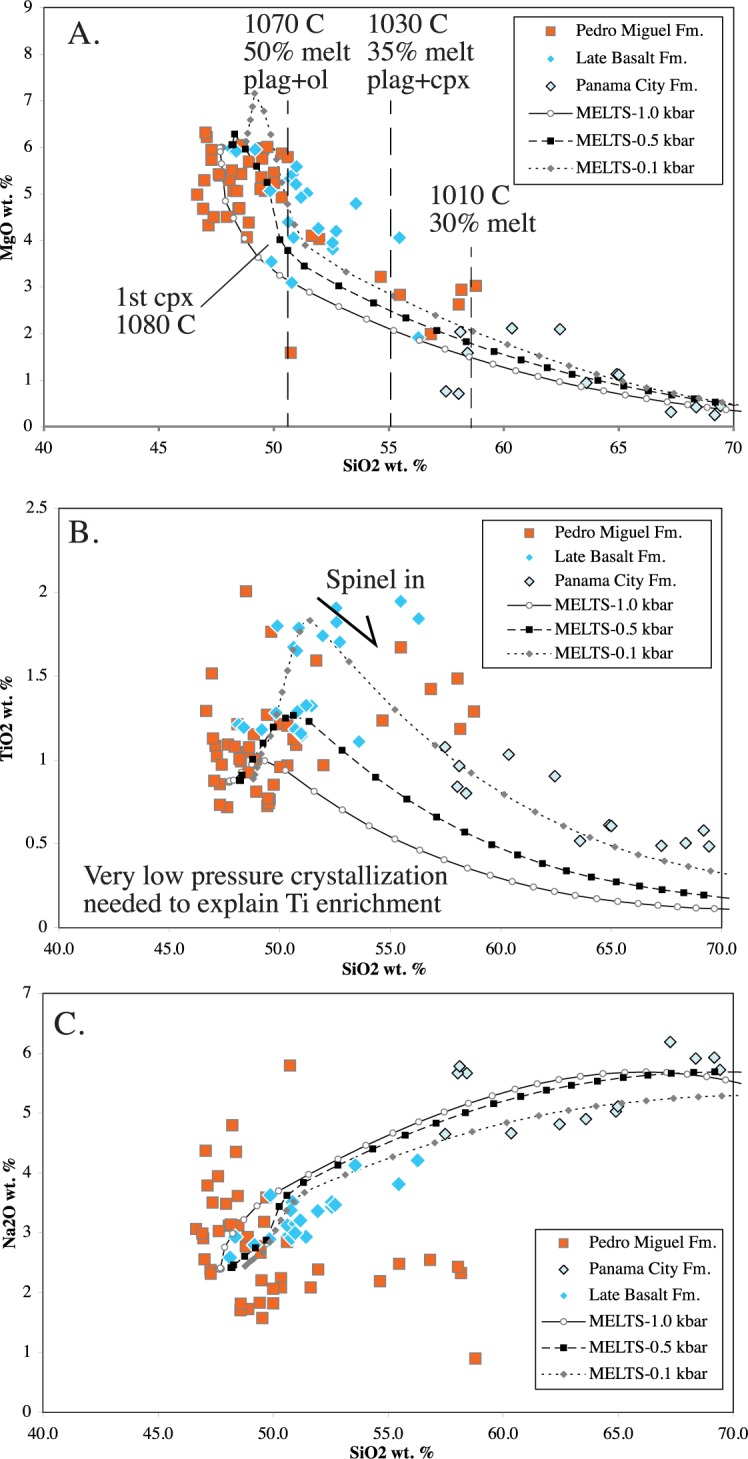
MELTS major element model of Miocene Canal volcanic rocks. Model runs at 0.1, 0.5 and 1.0 kbar are depicted. Liquidous temperatures are between 1190 and 1100°C. Most Pedro Miguel analyses can be reproduced with melt fractions greater than 0.5, however the most silicic Pedro Miguel rocks require melt fractions of 0.3 and the Panama City Formation rocks require melt fractions less than 0.15, which we consider to be physically unrealistic. A) MgO vs SiO_2_ wt. %, B) TiO_2_ vs SiO_2_ wt. %, C) Na_2_O vs SiO_2_.

Major element evolution has been modeled using the MELTS program of Ghiorso and Sack [42] and Asimow and Ghiorso [43] (Fig 19). A variety of starting compositions were tried, but Pedro Miguel Formation lavas with approximately 6 MgO wt. % worked best. Sample 070011 was used as the starting composition. 3 wt. % H_2_O was added to the analyzed composition for all model runs to create water saturated conditions. All model runs decreased in temperature from 1300°-700°C in 10° increments. In terms of pressure, models were run at 1.0, 0.5 and 0.1 kbar. QFM and NiNiO oxygen fugacity buffers were tried. In general QFM better reproduced the composition of the Pedro Miguel and Late basalt Formations, although NiNiO fugacities produce more accurate Al_2_O_3_ compositions. Overall, crystallization pressure appears to be the most significant physical variable, and in particular pressures below 0.5 kbar are needed to reproduce the diagnostic FeO* and TiO_2_ enrichments observed in the Late Basalt Formation.

Variation in crystallization pressure influences liquidus temperature and the order in which specific minerals form. The highest pressure runs had the lowest liquidus temperature with the 1.0 kbar run first crystalizing olivine at 1100°C, however olivine is a minor phase (< 1%) with significant clinopyroxene (10.5 g) and plagioclase (3.5 g) crystallizing by 1080°C. The lowest pressure (0.1 kbar) runs had a liquidus of 1190°C with anorthitic plagioclase being the initial phase. Also, significant plagioclase crystallization (13.8 g) occurs previous to the onset of olivine and clinopyroxene crystallization at 1150–40°C. Plagioclase remains by at least a three to one margin the most abundant phase in the low pressure run.

Crystallization pressure also significantly controlled the amount of water in the melt. At 1.0 kbar, the initial melt contained 2.9 wt. % H_2_O, whereas at 0.5 kbar and 0.1 kbar the amount of water in the initial melt decreased to 1.95 and 0.79 wt. %, respectively. In each case, the remaining water formed a second fluid phase along with the melt. Also, water in the melt phase increased during fractionation, but did so the least in the lowest pressure run (0.1 kbar).

Model runs were also conducted with lower amounts of initial water (e.g. 1.0 kbar with 0.8 wt. % H_2_O). These higher pressure / low water runs partially reproduced the characteristics of the 0.1 kbar run above (e.g. TiO_2_ enrichment during crystallization), but do not fit the data as well. Overall, low-pressure runs with significant excess water (but with low H_2_O content in the melt) best reproduced observed geochemical data.

In thin section, plagioclase is the first phase to crystallize in the Late Basalt Formation, and the low-pressure MELTS run reproduces this characteristic. The early and dominant crystallization of plagioclase is also a significant feature in reproducing the observed iron and titanium enrichments. Removal of minerals that lack iron and titanium drive the remaining melt concentrations upward and so reproduce the enrichment trends. This is a key characteristic of tholeiitic rocks [37]. Crystallized olivine is dominantly forsteritic (76%), and the clinopyroxene has significant diopside and clinoenstite components with only minor hedenbergite. Both are dominantly magnesian. Reduction of Fe and Ti peaks occurs with the crystallization of spinel that contains significant magnetite (Fe) and ulvospinel (FeTi) components. Higher-pressure model runs do not reproduce the iron and titanium enrichments observed in the data.

The above MELTS modeling suggest that most Pedro Miguel and Late Basalt Formation rocks formed by fractional crystallization of less than 50%. Individual samples fall nearly continuously along liquid lines of descent until approximately 50% crystallization is reached. Samples that correspond to greater degrees of fractionation exist, but are rare. In general, the Late Basalt Formation appears to have reached somewhat higher degrees of fraction. Silicic sub-units within the Pedro Miguel Formation (e.g. maroon ash) require up to 70% fractionation, but such units are small in volume. The largest group of silicic rocks comes from the Las Cascadas and Panama City Formations. These rocks can also be described by the above MELTS models, but require very high degrees of fractionation. To reach the most silicic end members of the Panama City Formation requires > 85–90% fractionation. Such high degrees of fractionation are not realistic due to significant increases in magma viscosity at low melt fractions (e.g. [44]). Overall, MELTS major element fractional crystallization models well characterize geochemical variations within the Pedro Miguel and Late Basalt Formations, but the extreme levels of fractional needed to reach the most silicic units (e.g. Panama City Formation) suggest an additional petrologic mechanism is needed to generate the complete range of observed compositional variability.

#### Trace element models

Full spectrum trace element models were produced to place constraints on the petrologic evolution of the Canal volcanic rocks. Distribution coefficients are from the Geochemical Earth Reference Model website [45] and Rollinson [46] (S2 Table). Mineral phases were determined by thin section analysis of samples from the various units. The minerals used in our preferred model include: plagioclase (69.5%), clinoproxene (23.2%), illmenite/magnetite (6.9%), and olivine (0.4%). There is also some indication in the models that sphene / allanite may be present in trace amounts, as relatively large high field strength element distribution coefficients are needed to make the model reproduce the data, but as these trace minerals were not observed in thin section, they were not explicitly included in the model.

**Fig 20:**
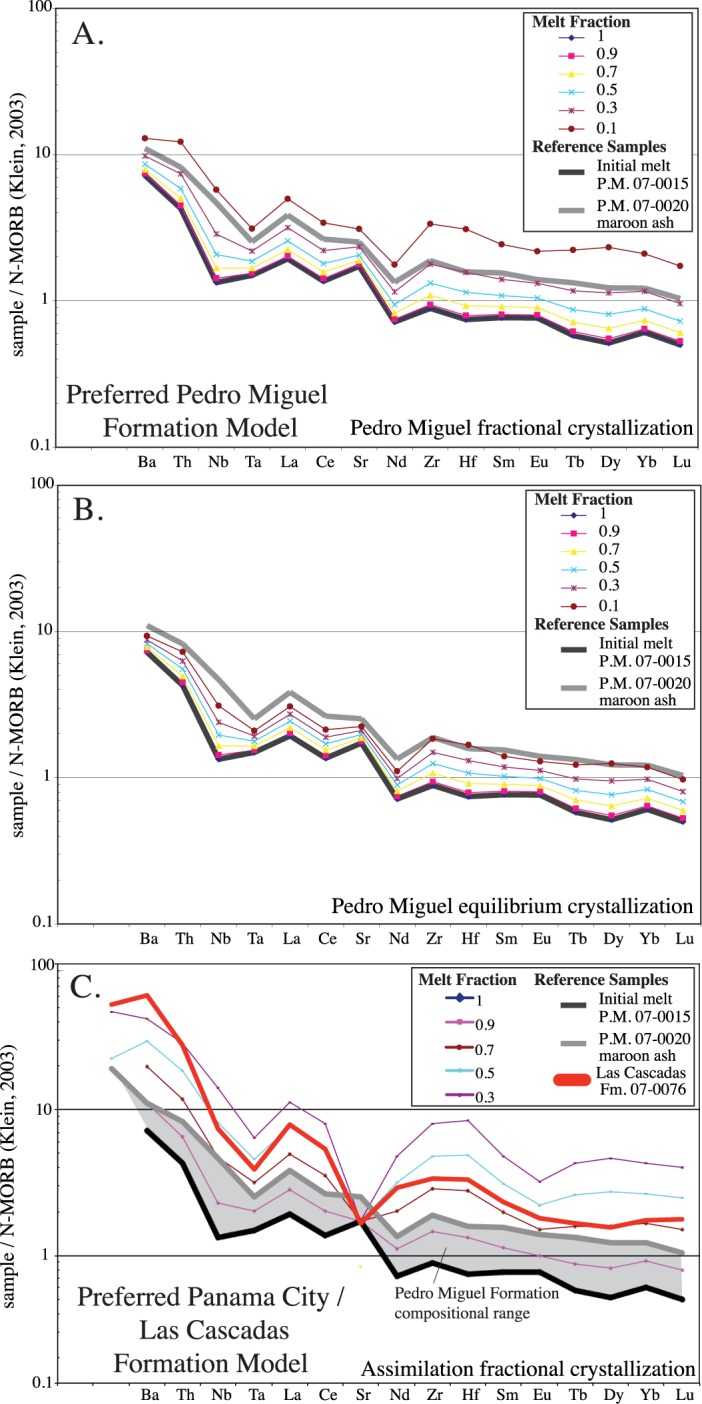
Trace element models. A) Fractional crystallization model. This model best reproduces the observed range of Pedro Miguel Formation compositions with the most silicic compositions reached at melt fractions of 0.3. B) Equilibrium crystallization model. This model also reproduces Pedro Miguel compositions, but require lower melt fractions. C) Assimilation fractional crystallization model. This model can reproduce trace element compositions from the most silicic Canal volcanic units (e.g. Las Cascadas Formation) using a Late Basalt Formation assimilant.

Fractional crystallization, equilibrium crystallization, and assimilation fractional crystallization models were used to determine which best fit the observed data (Fig 20). Starting compositions from the Bas Obispo, Pedro Miguel and Late Basalt Formations were used to see which could best reproduce more silicic Canal volcanic rocks. Sample 070015 was found to work best as a starting composition for generating more silicic Pedro Miguel Formation end members via fractional crystallization (Fig 20A). The Pedro Miguel maroon ash sub-unit is used as a silicic reference in the models. The initial composition sample 070015 is a basaltic pyroclastic rock with 5.42 MgO wt. % and an Mg # of 56.3. Pedro Miguel lavas were also tried, but did not work as well. Bas Obispo Formation basaltic pyroclastic rocks were tried but the fit was even worse than the Pedro Miguel lavas. Equilibrium crystallization models also reproduced the silicic Pedro Miguel compositions, but at low melt fractions (e.g. F = 0.1) (Fig 20B), and so were deemed not realistic. Overall, fractional crystallization models can reproduce the range of compositions observed within the Pedro Miguel and Late Basalt Formations with a maximum of approximately 70% fractionation (F = 0.3). This amount of fractional crystallization is high, but the total volume of silicic rocks within these units is low. Also, high temperature basaltic melts have relatively low viscosities and are therefore amenable too higher degrees of fractionation [47]. Finally, the degree of fractional crystallization present in the trace element models is similar to the major element MELTS models.

Pure fractional crystallization models do not reproduce the trace element patterns observed in the large volume silicic units such as the Las Cascadas and Panama City Formations. However, assimilation fractional crystallization models reproduce observed data at melt fractions of F = 0.7–0.5 and assimilation to fractionation ratios of r = 0.5. The assimilant used was the most silicic Late Basalt Formation rock (070079). This sample has a large negative Sr anomaly that is also present in the Las Cascadas and Panama City Formation data, but is not common in most Panamanian arc rocks. If the assimilant does not have the large negative Sr anomaly, the model does not accurately describe the data. We consider this to be a type of “self assimilation” in which initial Late Basalt Formation rocks are partially assimilated via subsequent intrusions. Magma mingling structures observed in certain Panama City Formation intrusions (Fig 8) are evidence that such internal digestion of earlier volcanic products may be a significant process. Overall, assimilation fractional crystallization models reproduce observed trace element patterns in the silicic Las Cascadas and Panama City Formations using Pedro Miguel starting compositions, and therefore tie the various Miocene Canal volcanic units together.

## Discussion

Overall, this paper has three main goals. They are: 1) To produce a quantitative petrologic model of the Canal region volcanic rocks, 2) To interpret the observed volcanic and tectonic structures in the area, and 3) to determine how goals 1 and 2 are inter-related and influenced by larger-scale tectonic changes in the Panama arc.

### Petrologic model of the Panama Canal volcanic rocks

The largest petro-tectonic distinction within the Canal volcanic rocks occurs at the Oligocene-Miocene boundary with Bas Obispo Formation and older arc rocks being dominated by amphibole fractionation processes that occur at mid-crustal depths [34]. In contrast, Miocene Canal volcanic rocks of diverse major element compositions share a common depletion in hydrous fluids and fluid mobile elements and have a reduced subduction signature with a significantly lessened negative Ta anomaly. Miocene basaltic and silicic end members also plot in extensional fields on tectonic discrimination diagrams (Fig 17). The full range of Pedro Miguel and Late Basalt Formation rocks can be reproduced in trace and major element models via pure fractional crystallization that occurs at very shallow (0.5–0.1 kbar) pressures (Figs 19 and 20). Within individual Pedro Miguel volcanic edifices basaltic and andesitic rocks alternate. This suggests that multiple episodes of magma recharge and eruption occurred within each body. Within each eruptive sequence more silicic rocks occur first. This suggests that the underlying magma chamber was underwent continuous fractionation, but was only periodically tapped during eruption. During each eruptive sequence, the top and therefore the most silicic portion of the magma chamber erupted first, followed by less evolved lavas. Such a mechanism would reproduce the observed volcanic stratigraphy within the Pedro Miguel Formation, and matches in detail what is observed at Hodges Hill.

The most silicic Miocene units include the Las Cascadas and Panama City Formations, and the trace element signature of these rocks cannot be reproduced using pure fractional crystallization models. However as indicated above, assimilation fractional crystallization models do successfully reproduce Las Cascadas and Panama City Formation rocks at reasonable melt fractions (Fig 20C). Therefore, we suggest that assimilation fractional crystallization (AFC) processes are responsible for the creation of the most silicic Canal volcanic rocks.

The geochemical models discussed above do a good job of explaining petrologic variability within the Miocene Canal volcanic rocks, but the do not really address the question of why they are different (e.g. hotter dryer melts with shallower fractionation depths) from those in the Oligocene. Rooney et al. [34] proposed arcs produce melts with both wet and dry fractionation trends and that the Las Cascadas Formation is simply part of the dry fractionation melt series. Rooney et al. [34] also suggested that the Las Cascadas Formation rocks were more silicic because they were part of the dry trend, but they did not examine basaltic Miocene rocks such as the Pedro Miguel Formation.

Zimmer et al. [48] described a relationship between calc-alkaline and tholeiitic rock series and the amount of water the magmas contain. They analyzed this relationship by calculating a tholeiitic index (THI) and then quantifying how the tholeiitic index varies with respect to the amount of independently measured water in a melt. THI is the ratio of FeO* wt. % at MgO values of 4.0 and 8.0 wt. %, respectively. The following formulas allow the amount of water in a melt to be calculated from major element data:

$$THI=\frac{FeO_{4.0}^{*}}{FeO_{8.0}^{*}}$$ (1)

$$H2O\;wt\;\%=exp\left[\frac{\left(1.26-THI\right)}{0.32}\right]$$ (2)

**Fig 21:**
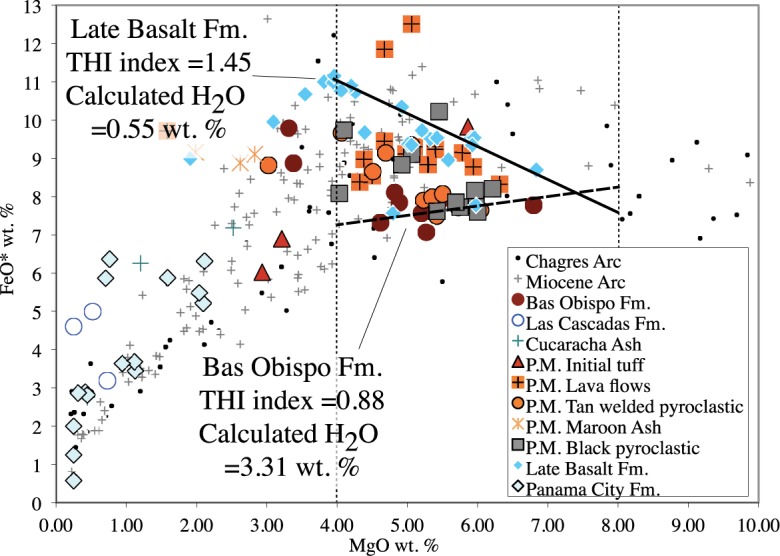
FeO* wt. % vs MgO wt. % used to calculate the tholeiitic index (THI) after Zimmer et al. [48]. The THI can be used to calculate the amount of water in a melt of a given composition. The Bas Obispo Fm. has a THI of 0.88 and a calculated H_2_O wt. % of 3.31, whereas the Late Basalt has a THI of 1.45 and a calculated H_2_O wt. % of 0.55, indicating that the Canal volcanic rocks became much dryer and more tholeiitic in the Miocene.

Applied to the Canal volcanic rocks these yield a THI = 1.45 and H_2_O_calc_ wt. % = 0.55 for the Late Basalt Formation and values of THI = 0.88 and H_2_O_calc_ wt. % = 3.31 for the Bas Obispo Formation (Fig 21). The Pedro Miguel Formation rocks lie between the above units. Overall, the calculated H_2_O_calc_ wt. % values are consistent with the idea of a shift to dryer more tholeiitic magmatism in the Miocene.

Finally, Zimmer et al. [48] present a list of THI index values and water contents for various volcanic systems around the world. The Bas Obispo Formation values are consistent with those derived from subduction settings including those from: Alaska, Central America, the Cascades and Japan. In contrast, THI indices similar to the Late Basalt Formation (1.45) are only found in back-arc basin, mid-ocean ridge and ocean island settings. However, THI indices from back-arc basins are most similar and include those from: the Mariana Trough, the Lau, Woodlark and North Fiji Basins.

There are several observations that suggest the change from wet to dry arc magmatism was not part of normal evolution in arc magmatism, but instead was forced into this mode by specific tectonic events. First is the change observed using tectonic discrimination diagrams in which both basaltic and silicic Miocene rocks plot in extensional fields. Second, the Miocene volcanic rocks have a decreased subduction signature (smaller Ta anomaly) compared to adjacent Oligocene rocks. Third, structural mapping shows abundant normal faulting and shows that the Canal itself sits within a structural graben that likely provided “accommodation space” for the volcano-sedimentary units of the Canal Basin. Collectively these observations suggest the onset of an extensional event at the Oligocene-Miocene boundary. In this interpretation, extension thinned the crust and caused upwelling in the underlying asthenosphere. Continued decompression melting of the asthenosphere and magma ascent to very shallow levels, caused the resulting melts to dry out. Such a model would account for what is observed in the Canal region and what is required by geochemical models presented here.

Magmatism sourced in the Canal region ends earlier than volcanic activity outside the region (e.g. El Valle, [22]). The youngest unit we observe along the Canal is the Late Basalt Formation, and while not dated, it is in stratigraphic continuity with the Pedro Miguel Formation and so has a similar if somewhat younger age. Conservatively, it can be stated that the Late Basalt Formation is older than 15 Ma, and since this time there has been no active magmatism along the Panama Canal. Younger Canal rocks such as the Gatun Formation on the Caribbean coast contain ash layers [27], but these are sourced elsewhere (most likely El Valle). We postulate that the same petrologic mechanism that led to the distinct extensional geochemical signatures of the Miocene Canal volcanic rocks also led to the early cessation of magmatism in this region. Our preferred petrologic mechanism for Canal volcanism is decompression melting of the sub-arc mantle due to lithospheric extension. Partial melting of the sub-arc mantle can only occur for so long without fluid replenishment due to sub-arc mantle corner flow. If the extension and fracturing of the Isthmus [16] inhibited corner flow, then the sub-arc mantle beneath the Canal region would eventually become infertile and restitic. Such a result is consistent with the observation that Canal magmatism ends by approximately 15 Ma, but continues in other parts of the Panama arc.

### Interpretation of mapped volcanic structures

**Fig 22:**
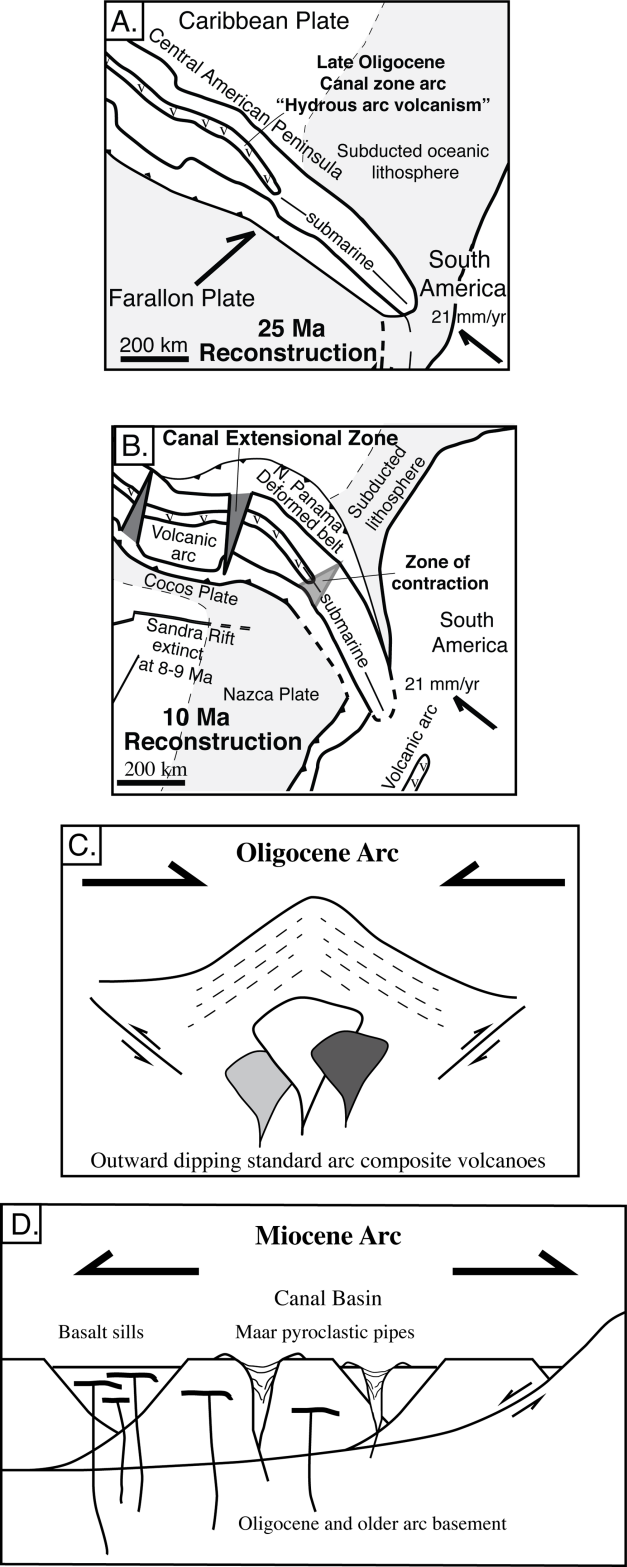
Schematic model of Canal region magmatic variation due to changing tectonic conditions. A. Map view tectonic model of Panama in the Oligocene (modified from Farris et al. [16]). This is previous to the collision with South America. B. Map view tectonic model of the Panama arc in the Miocene, after collision with South America. Note the existence of the Canal extensional zone. C. Oligocene arc. Magmatism at this time is defined by deeper hydrous amphibole bearing magmas that form standard arc composite volcanoes underlain by plutonic systems. D. Miocene arc. Volcanism is heavily influenced by the onset of arc perpendicular extension in the Canal region. Magmatism is hot, shallow, anhydrous and occurs dominantly as basaltic sills and inward dipping maar pyroclastic pipes that intrude the Canal Basin.

Overall, detailed geologic mapping along the Panama Canal has defined a sequence of large basaltic sills (Late Basalt Fm.) and inward dipping pyroclastic edifices (Pedro Miguel Fm.). The inward dipping structures are interpreted as volcanic maars. In volcanic arcs such modes of volcanic intrusion and extrusion are not common, but have been described in extensional settings such as the Auckland volcanic field in New Zealand [49, 50, 51]. Volcanic arcs typically produce plutonic intrusions of gabbro to granodiorite overlain by outward dipping composite volcanoes. However, sill and maar intrusive and extrusive forms are most common in large igneous provinces such as the Karoo of South Africa, the Ferrar Supergroup in Antarctica, and the Siberian Traps [52, 53, 54, 55, 56]. It is our interpretation that the volcanic forms observed along the Panama Canal formed are due to the physical properties of the intruding magmas. That is, that the observed change from hydrous arc magmas in the Oligocene to dry, hot extensional magmatism in the Miocene also corresponded to a change in the type of constructed magmatic structures (Fig 22).

Lorenz [57] and Lorenz and Kurszlaukis [58] define maar-diatreme volcanism as forming from an explosive eruption that creates a pipe-like crater. Surrounding the crater, if preserved, will be a tephra ring. The interior of the pipe is subsequently filled with additional inward dipping tephra and pyroclastic deposits. Any remaining accommodation space at the top of the crater can be subsequently filled with post eruptive sediments or additional near horizontal volcanic deposits if volcanism remains active in the nearby area. Such a volcanic stratigraphy matches quite well what is observed in the Pedro Miguel Formation, particularly at Hodges Hill.

Maar-diatreme volcanism is often interpreted to result from phreatomagmatic processes [57]. For example, if an underlying feeder dike or sill encounters significant near surface ground water, explosive volcanism can result creating the observed volcanic pipe. Multiple to hundreds of individual explosions can occur to excavate the initial pipe, depending on exactly how ground water interacts with the magmatic system. Lorenz and Kurszlaukis [58] suggest that the explosive eruptions progressively excavate the pipe downwards and that within the root zone a breccia of shatter host rocks and eruptive magmas develops. In the Panama Canal examples presented here, roots of the maar complexes are not exposed. However, the middle eruptive sequences at Hodges Hill contain tephra deposits composed of fragments of underlying welded pyroclastic rocks. This indicates that multiple explosive eruptions were required to build the observed structure.

### Interpretation of sub-volcanic features

Sub-volcanic and shallow level intrusive structures have also been studied in volcanic arcs, and we will now compare our observations along the Canal to an example of hydrous magmatism from the Sierra Nevada batholith in California. Sisson et al. [59] examined the 92 Ma gabbroic Onion Valley Sill complex and observed a sequence of over 300 m of hornblende gabbro and diorite sills that overlie an approximately 200 m thick cummulate body. The emplacement depth is estimated at between 1–2 kbar. This is deeper than the Canal basaltic sills, but is still a useful point of comparison for dry vs wet arc magmatism. Sisson et al. [59] state that the Onion Valley sills were emplaced at volatile saturation due to the presence of miarolitic cavities and estimate total dissolved water content at near 6 wt. %. This amount of water is much greater than was present in the Miocene Canal volcanic magmas, and approximately twice the calculated value for the Oligocene Bas Obispo Formation. Sisson et al. [59] also state that the high water content of the Onion Valley basaltic magmas significantly influenced their physical properties and in particular lowered the liquidus temperature and melt density. The estimated liquidus temperature of the Onion Valley magmas is approximately 1000°C. In comparison, MELTS modeling of the Pedro Miguel Formation suggests a liquidus temperature of between 1100–1190°C for crystallization pressures of 1.0–0.1 kbar, respectively. Also, the MELTS results agree with other temperature estimates for dry basaltic arc magmas (e.g. 1100°C, [60]). Therefore, the dry and shallow nature of the Pedro Miguel and Late Basalt Formations cause them to be 100–200°C hotter than older (Oligocene) hydrous arc melts. This likely played a significant role in the types of volcanic structures formed in that the hotter dryer melts led to more explosive ground water interactions and caused maar and sill structures to be dominant.

### Tectonic and structural implications

**Fig 23:**
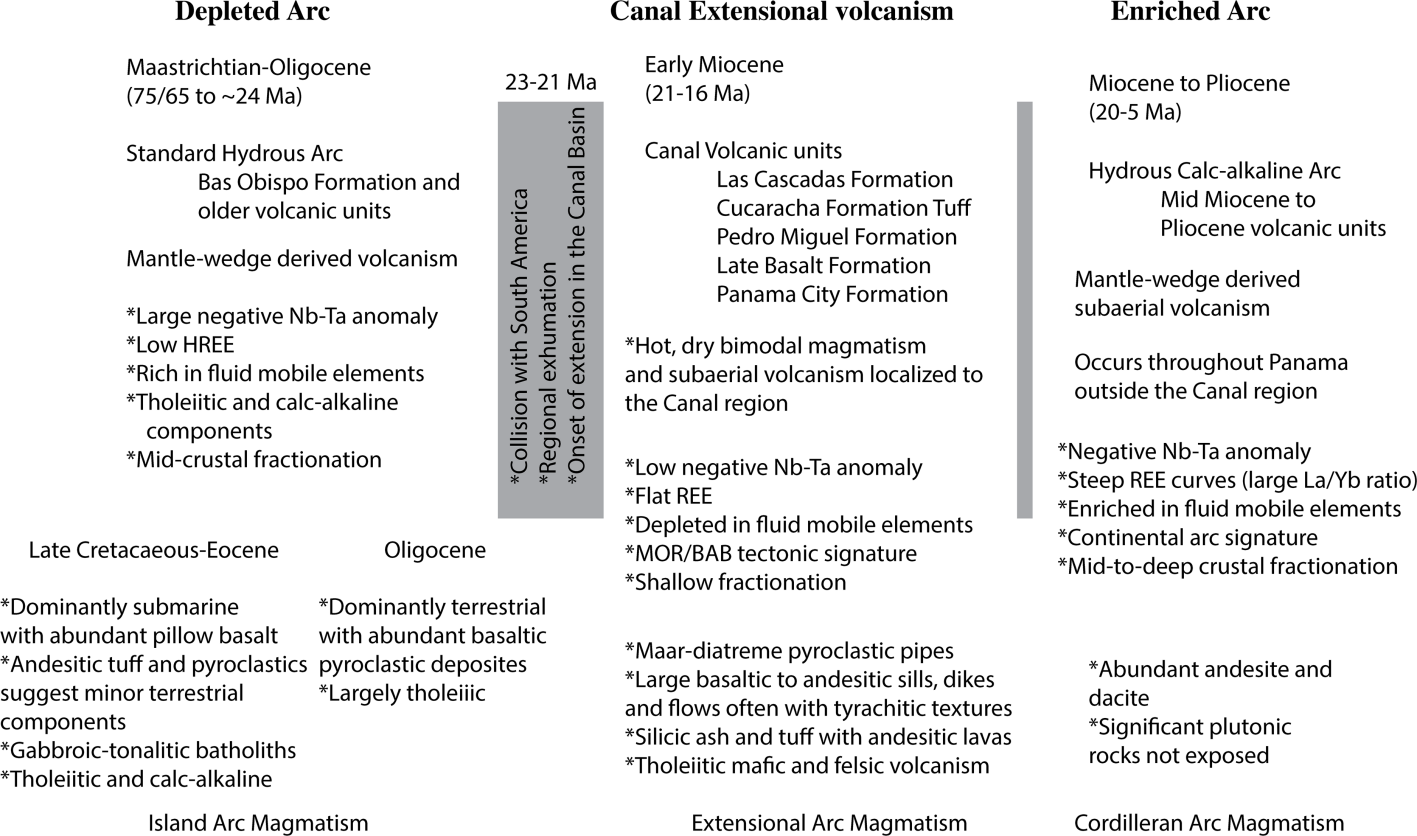
Summary of Panama arc evolution from initiation through Pliocene. The extensional Canal magmatism is unique throughout this period. Note this figure does not contain the modern adakitic magmatism that today dominates western Panama.

Overall, structural mapping and geochemical data indicate that Canal arc magmatism transitions from hydrous mantle-wedge derived volcanism to magmatism dominated by extensional characteristics at the Oligocene-Miocene boundary (Fig 23). Structural maps and cross-sections clearly show the existence of normal faults and that the Panama Canal itself exists in a small structural graben. The largest normal faults are Canal parallel / Isthmus perpendicular. This is the orientation of faults predicted in the Canal extensional zone of Farris et al. [16], however there are both Canal parallel and Canal perpendicular normal faults. Barat et al. [61] also produced a tectonic model that shows extension within the Canal region, but they indicate extension occurs as the result of step overs on a series of approximately N-S strike-slip faults, and that normal faults should be dominantly E-W. As mentioned above, the largest normal faults are roughly Canal parallel, or perpendicular to the orientation predicted in the Barat et al. [61] model. One explanation for observed normal fault orientations is as the Canal extensional zone opened due to fracturing and oroclinal rotation of the Isthmus during collision with South America [16], the extended crust, no longer attached to the coherent Panama block, collapsed northward towards the free surface of the Caribbean plate. This would produce dominantly E-W extension with a lesser component in the N-S direction, as observed.

The transition to hot-dry extensional volcanism in the Miocene strongly suggests the occurrence of kilometer-scale crustal thinning. However, normal faults mapped along the Canal itself, although numerous, are not large enough to accommodate lithospheric-scale extension. Gravity studies by Farris [62], Fowler [63] and Mynhier [64] suggest that larger detachment-scale normal faults exist and delineate a series of sub-basins within the Canal region. Overall, the mapping areas discussed in this paper represent small-scale intra-basin deformation, but the observed tectonic styles are representative of what exists on the scale of the entire Canal Basin.

Exhumation of basement massifs east of the Canal also yields information on the tectonic evolution of the Canal Basin itself. Low-temperature thermochronology data presented in Farris et al. [16], Montes et al. [33], and Ramírez et al. [65] indicate that exhumation of arc basement massifs east of the Canal occurred contemporaneously with the onset of extensional magmatism at the beginning of the Miocene. During the Miocene, the Canal Basin itself must have undergone subsidence as it was accumulating sediments at this time. However, (U-Th)/He apatite ages in the Cerro Azul and Rio Mamoni areas east of the Canal suggest exhumation between 10–25 Ma. In addition, these ages young westward toward the Canal region. One explanation is that arc basement rocks closest to the Canal basin are part of a Canal extensional zone rift flank and consequently experienced greater exhumation that continued until extension ceased.

Normal faults in our Canal mapping areas are cut by younger pre-dominantly left-lateral strike-slip faults (Figs 1, 9 and 13). Such an observation makes sense as modern tectonics of the Canal region are dominated by strike-slip faults such as the Rio Gatun and Pedro Miguel faults [66]. This indicates that the extensional regime described in this paper ended at some point and was replaced by strike-slip tectonics. The largest strike strike-slip faults along the Canal are perpendicular to it and also at a high angle to the active Pedro Miguel fault (Fig 1). The Pedro Miguel fault is right-lateral, whereas the Canal strike-slip faults are left-lateral suggesting they may be conjugates. Rockwell et al. [66] indicate slip-rates on the Pedro Miguel and Mira-Flores faults are on the order of 4–7 mm /yr. Total displacement along the Pedro Miguel fault is no more than 5–10 km given offset units (Fig 1), and therefore must be younger than 1–3 million years old. Other age constraints on the onset of strike-slip tectonics and the end of extension come from the northern Canal region. Young normal faults have been described in the Gatun and Chagres Formation (e.g. [67]), and the depositional age of the youngest Chagres Formation rocks are approximately 5.8 Ma. Overall, this suggests that extension in the Canal region persisted throughout the Miocene and ended between 5.8 and 3 Ma. However, extensional related magmatism as described in this paper likely ended by 15 Ma.

## Conclusions

1. Canal region volcanic rocks preserve a sharp transition from hydrous mantle wedge magmatism to hot dry bimodal tholeiitic volcanism across the Oligocene-Miocene boundary.
2. The transition from deeper hydrous to shallow dry arc magmatism is synchronous with formation of the Panama Canal basin and extensional faulting. The Panama Canal sedimentary basin sits within a fault bounded graben with the largest normal faults running parallel to its axis. However, the basin experienced a complex faulting environment with normal faults both parallel and perpendicular to its axis. Finally, normal faults are overprinted by a younger generation of strike-slip faults. Overall, the transition in arc chemistry was driven by the onset of extension at the Oligocene-Miocene boundary (21–25 Ma).
3. Geochemical modeling of major and trace elements indicate that the Pedro Miguel and Late Basalt Formations formed via shallow level fractional crystallization at pressures between 0.5–0.1 kbar and initial temperatures of 1100–1190°C. Modeling also suggests that the most silicic units such as the Las Cascadas and Panama City Formations formed via assimilation fractional crystallization. The lowest pressures indicate that very little exhumation has occurred since the Miocene.
4. The Miocene Canal volcanic rocks take several different physical forms. The Las Cascadas Formation and Cucaracha tuff are bedded volcanic layers within the Canal sedimentary basin. However, the Pedro Miguel, Late Basalt and Panama City Formations are in-part intrusive. The Pedro Miguel Formation forms a series of discrete inward-dipping maar-diatreme volcanic pipes that formed via multiple pyroclastic eruptions. The overlying, slightly more evolved Late Basalt Formation occurs primarily as large sills with extensive columnar jointing and surficial lava flows. Finally, the Panama City Formation occurs as dacitic plugs and lava flows.
5. The Pedro Miguel volcanic edifices contain multiple eruptive cycles that initiate with silicic pyroclastic deposits that subsequently become more mafic. Our interpretation is that such cycles are due to multiple episodes of intrusion into a continuously fractionating sub-volcanic magma chamber. Each intrusion triggers the eruption of the top and most silicic magmas followed by less evolved melts from the middle and bottom of the sub-volcanic chamber. Finally, interaction of the intruding magmas with Canal Basin ground water also played a significant role in the generation of basaltic pyroclastic eruptions.
6. The maar-diatreme and basaltic sill physical form of the Miocene Canal volcanic bodies is a characteristic of extensional arcs, however volcanic edifices of this type are most abundant in continental Large Igneous Provinces and rift environments. It is our interpretation that the change from hydrous to hotter and dryer magmatism across the Oligocene-Miocene boundary drove a change in magma emplacement style and volcanic edifice form. This suggests that the physical characteristics (e.g. temperature and water content) of the magma are a primary determinant in its petrologic and volcanologic evolution.
7. The extensional nature of Miocene Canal magmatism and its subsequent cessation by 15 Ma are related by the petrologic mechanism of mantle decompression melting caused by crustal extension. Continued partial melting led the Canal sub-arc mantle to become infertile and therefore caused cessation of locally sourced magmatism in the Canal region. However, extension continued until 6–3 Ma before being replaced with the modern strike-slip dominated tectonic regime.

## Supporting information

S1 Table Panama Canal volcanic rock major and trace element data. (XLSX)

S2 Table Canal volcanic rock trace element distribution coefficients. (XLSX)

## References

[1] Gill J., Orogenic andesites and plate tectonics. Berlin, Springer-Verlag, 1981, 390 p.

[2] Taylor S.R., The geochemical evolution of the continental crust. Reviews of Geophysics, 1995, v. 33, p. 241–265,

[3] Rudnick R.L., Making continental crust. Nature, 1995, v. 378, p. 571–578,

[4] Kelemen P. B., Hanghøj K. & Greene A. R., One view of the geochemistry of subduction-related magamatic arcs, with emphasis on primitive andesite and lower crust In: Rudnick R. L. (ed.) The Crust, Vol. 3, Holland H.D. and Turekian K. K. (eds) Treatise on Geochemistry 2003, Oxford: Elsevier– Pergamon, pp. 593–659.

[5] Gazel E., Hayes J. L., Hoernle K., Kelemen P., Everson E., Holbrook W. S., Hauff F., van den Bogaard P., Vance E. A., Chu S., Calvert A. J., Carr M. J., and Yogodzinski G. M., Continental crust generated in oceanic arcs. Nature Geoscience, 2015, v. 8, no. 4, p. 321–327.

[6] Peacock SM. Are the lower planes of double seismic zones caused by serpentine dehydration in subducting oceanic mantle?. Geology. 2001 4 1;29(4):299–302.

[7] Tatsumi Y., The Subduction factory: How it operates in the evolving Earth. GSA Today, 2005, v. 15, no. 7,

[8] England P., Engdahl R., and Thatcher W., Systematic variation in the depths of slabs beneath arc volcanoes: Geophysical Journal International, 2004, 156,

[9] Syracuse E.M., Abers G.A., Global compilation of variations in slab depth beneath arc volcanoes and implications: Geochemistry Geophysics Geosystems, 2006, vol. 7, no. 5,

[10] Perfit M.R., Gust D.A., Bence A.E., Arculus R.J. and Taylor S.R. Chemical characteristics of island arc basalts: implications for mantle sources: Chemical Geology, 1980, 30, 1–29.

[11] Lee J., Stern R.J., and Bloomer S.H., Forty million years of magmatic evolution in the Mariana arc: The tephra glass record: Journal of Geophysical Research, 1995, vol. 100, no. B9, p. 17,671–17,687.

[12] Arculus, R. J., J. B. Gill, H. Cambray, W. Chen, and R. J. Stern, Geochemical evolution of arc systems in the western Pacific: The ash and turbidite record recovered by drilling, in Active Margins and Marginal Basins of the Western Pacific, Geophys. Monogr. Ser., 1995, vol. 88, edited by B. Taylor and J. Natland, pp. 45–65, AGU, Washington, D. C.,

[13] Hochstaedter A. G., Gill J. B., Taylor B., Ishizuka O., Yuasa M., and Monta S., Across-arc geochemical trends in the Izu-Bonin arc: Constraints on source composition and mantle melting, J. Geophys. Res., 2000, 105(B1), p. 495–512,

[14] Smith I.E.M., and Price R.C.,The Tonga–Kermadec arc and Havre–Lau back-arc system: Their role in the development of tectonic and magmatic models for the western Pacific: Journal of Volcanology and Geothermal Research, 2006, 156, p. 315–331.

[15] Saginor I., Gazel E., Condie C., and Carr M. J., Evolution of geochemical variations along the Central American volcanic front: Geochemistry, Geophysics, Geosystems, 2013, v. 14, no. 10, p. 4504–4522.

[16] Farris D.W., Jaramillo C., Bayona G., Restrepo S.A., Montes C., Cardona A., Mora A., Speakman R.J., Glasscock M.D., and Valencia V., Fracturing of the Panamanian Isthmus during initial collision with South America: Geology, 2011, v. 39, no. 11, p. 1007–1010,

[17] Buchs D.M., Arculus R., Baumgartner P. O., Baumgartner-Mora C., Ulianov A. Late Cretaceous Arc development on the SW margin of the Caribbean Plate: insights from the Golfito, Costa Rica, and Azuero, Panama, complexes: Geochemistry, Geophysics, Geosystems, 2010, 11, 7, Q07S24.

[18] Wörner G, Harmon RS, Wegner W. Geochemical evolution of igneous rocks and changing magma sources during the formation and closure of the Central American land bridge of Panama. Geological Society of America Memoirs. 2009, 6 1;204:183–96.

[19] Wegner W, Wörner G, Harmon RS, Jicha BR. Magmatic history and evolution of the Central American Land Bridge in Panama since Cretaceous times. Geological Society of America Bulletin. 2011, 3 1; v. 123, no. 3–4, p.703–724.

[20] Whattam S.A., Montes C., McFadden R.R., Cardona A., Ramirez D. and Valencia V., Age and origin of earliest adakitic-like magmatism in Panama: Implications for the tectonic evolution of the Panamanian magmatic arc system: Lithos, 2012, 142, pp.226–244.

[21] Defant M. J., Clark L. F., Stewart R. H., Drummond M. S., de Boer J. Z., Maury R. C., Bellon H., Jackson T. E., and Restrepo J. F., Andesite and dacite genesis via contrasting processes: The geology and geochemistry of El Valle Volcano, Panama, Contrib. Mineral. Petrol., 1991,106, p. 309–324.

[22] Defant M. J., Jackson T. E., Drummond M. S., de Boer J. Z., Bellon H., Feigenson M. D., Maury R. C., and Stewart R. H., The geochemistry of young volcanism throughout western Panama and southeastern Costa-Rica: An overview. J. Geol. Soc. London, 1992, 149, p. 569–579.

[23] Gazel E., Hoernle K., Carr M. J., Herzberg C., Saginor I., den Bogaard P. v., Hauff F., Feigenson M., and Swisher C., Plume–subduction interaction in southern Central America: Mantle upwelling and slab melting. Lithos, 2011, v. 121, no. 1–4, p. 117–134.

[24] Abratis M., and Wo¨rner G., Ridge collision, slab-window formation, and the flux of Pacific asthenosphere into the Caribbean realm. Geology, 2001, 29, p. 127–130.

[25] Goss A.R., and Kay S.M., Steep REE patterns and enriched Pb isotopes in southern Central American arc magmas: Evidence for forearc subduction erosion?. Geochemistry, Geophysics, Geosystems, 2006, vol. 7, no. 5, 20 p.

[26] Hidalgo, P.J., Vogel, T.A., Rooney, T.O., Currier, R.M., and Layer, P.W., Origin of silicic volcanism in the Panamanian arc: evidence for a two-stage fractionation process at El Valle volcano: Contributions to Mineralogy and Petrology, 2011,

[27] Rooney T.O., Morell K.D., Hidalgo P. and Fraceschi P., Magmatic consequences of the transition from orthogonal to oblique subduction in Panama: Geochemistry, Geophysics, Geosystems, 2015, 16(12), pp.4178–4208.

[28] MacDonald, D.F., Some engineering problems of the Panama canal in their relation to geology and topography: Department of the Interior, Bureau of Mines, 1915, 88p.

[29] Lutton, R.J. and Banks, D.C., Study of clay shale slopes along the Panama Canal, Report 1: U.S. Army Engineer Waterways Experiment Station Technical Report S-70-9, 1970, 326 p.

[30] Stewart, R.H., and J.L., Stewart, Geologic map of the Panama Canal and vicinity, Republic of Panama, United States Geological Survey, Miscellaneous investigations series map I-1232, 1980.

[31] Kirby M.X., Jones D.S., MacFadden B.J.,Lower Miocene Stratigraphy along the Panama Canal and Its Bearing on the Central American Peninsula: PloS ONE, 2008, 3(7): e2791 doi: 10.1371/journal.pone.0002791 18665219 10.1371/journal.pone.0002791 PMC2464738

[32] MacFadden B.J., Kirby M.X., Rincon A., Montes C., Moron S., Strong N., and Jaramillo C., Extinct peccary ‘‘Cynorca” Occidentale (Tayassuidae, Tayassuinae) from the Miocene of Panama and correlations to North America: Journal of Paleontology, 2010, v. 84, no. 2, pp. 288–298.

[33] Montes C., Cardona A., Bayona G., MacFadden R., Buchs D.M., Moron S.E., Silva C.A., Hoyos N., Restrepo-Moreno S., Ramirez D.A., Wilson J., Ortiz J., Farris D.W., Jaramillo C., Valencia V., Bryan J., and Flores J.A., Evidence for middle Eocene and younger land emergence in central Panama: Implications for Isthmus closure. Geological Society of America Bulletin, 2012, 20 p.,

[34] Rooney T.O., Franceschi P., and Hall C.M., Water-saturated magmas in the Panama Canal Region: a precursor to adakite-like magma generation?. Contributions to Mineralogy and Petrology, 2010,

[35] Bloch JI, Woodruff ED, Wood AR, Rincon AF, Harrington AR, Morgan GS, Foster DA, Montes C, Jaramillo CA, Jud NA, Jones DS. First North American fossil monkey and early Miocene tropical biotic interchange. Nature. 2016 5 12;533(7602):243–6. doi: 10.1038/nature17415 27096364 10.1038/nature17415

[36] MacFadden B.J., Block J.I., Evans H., Foster D.A., Morgan G.S., Rincon A., and Wood A.R., Temporal Calibration and Biochronology of the Centenario Fauna, Early Miocene of Panama: The Journal of Geology, 2014, vol. 122, p. 113–135.

[37] Grove T.L., and Baker M.B., Phase equilibrium controls on the tholeiitic versus calc- alkaline differentiation trends: Journal of Geophysical Research, 1984, v. 89, p. 3253–3274.

[38] Shervais J.W., Ti-V plots and the petrogenesis of modern and ophiolitic lavas: Earth and Planetary Science Letters, 1982, 59, p. 101–118.

[39] Wood D.A., The application of Th-Hf-Ta diagram to problems to tectonomagmatic classificationand to establishing the nature of crustal contamination of basaltic lavas of the British Tertiary volcanic province: Earth and Planetary Science Letters, 1980, 50, p. 11–30.

[40] Pearce J.A., Harris N.B.W., and Tindle A.G., Trace element discrimination diagrams for the tectonic interpretation of granitic rocks: Journal of Petrology, 1984, 25, p. 956–983.

[41] Pearce J.A. and Peate D.W., Tectonic implications of the composition of volcanic arc magmas: Annual Review of Earth and Planetary Sciences, 1995, vol. 23, p. 251–285.

[42] Ghiorso Mark S., and Sack Richard O.,Chemical Mass Transfer in Magmatic Processes. IV. A Revised and Internally Consistent Thermodynamic Model for the Interpolation and Extrapolation of Liquid-Solid Equilibria in Magmatic Systems at Elevated Temperatures and Pressures. Contributions to Mineralogy and Petrology, 1995, 119, p. 197–212.

[43] Asimow P.D., Ghiorso M.S., Algorithmic Modifications Extending MELTS to Calculate Subsolidus Phase Relations. American Mineralogist, 1998, 83, p. 1127–1131.

[44] Arzi A. A., Critical phenomena in the rheology of partially melted rocks: Tectonophysics, 1978, 44, no. 1–4, p. 173–184.

[45] https://earthref.org/KDD/.

[46] Rollinson H., Using geochemical data: Evaluation, presentation, interpretation: Singapore, Longman Group, 1993, 352 p.

[47] Giordano D., Russell J.K., and Dingwell D.B.,Viscosity of magmatic liquids: A model: Earth and Planetary Science Letters, 2008, 271,

[48] Zimmer M.M., Plank T., Hauri E.H., Yogodzinski G.M., Stelling P., Larsen J., Singer B., Jicha B., Mandeville C. and Nye C.J., The role of water in generating the calc-alkaline trend: new volatile data for Aleutian magmas and a new tholeiitic index: Journal of Petrology, 2010, p. 2411–2444.

[49] Houghton B.F., Wilson C.J.N. and Smith I.E.M.,Shallow-seated controls on styles of explosive basaltic volcanism: a case study from New Zealand. Journal of Volcanology and Geothermal Research,1999, 91(1), pp.97–120.

[50] Cassidy J., France S.J. and Locke C.A., Gravity and magnetic investigation of maar volcanoes, Auckland volcanic field, New Zealand: Journal of volcanology and geothermal research, 2007, 159, pp.153–163.

[51] Molloy C., Shane P. and Augustinus P., Eruption recurrence rates in a basaltic volcanic field based on tephra layers in maar sediments: implications for hazards in the Auckland volcanic field. Geological Society of America Bulletin, 2009, 121(11–12), pp.1666–1677.

[52] White J.D.L., and McClintock M.K., Immense vent complex marks flood-basalt eruption in a wet, failed rift: Coombs Hills, Antarctica: Geology, 2001, vol. 29, no. 10, p. 935–938.

[53] Jamtveit B, Svensen H, Podladchikov YY, Planke S. Hydrothermal vent complexes associated with sill intrusions in sedimentary basins. Physical geology of high-level magmatic systems. 2004 1 1;234:233–41.

[54] Svensen H., Planke S., Malthe-Sorenssen A., Jamtveit B., Myklebust R., Eidem T.R., and Rey S.S., Release of methane from a volcanic basin as a mechanism for initial Eocene global warming: Nature, 2004, vol. 429, p. 542–545. doi: 10.1038/nature02566 15175747 10.1038/nature02566

[55] Svensen H., Jamtveit B., Planke S., Chevallier L., Structure and evolution of hydrothermal vent complexes in the Karoo Basin: South Africa. J. Geol. Soc., 2006,163, 671–682.

[56] Svensen H., Planke S., Polozov A.G., Schmidbauer N., Corfu F., Podladchikov Y.Y., Jamtveit B., Siberian gas venting and the end-Permian environmental crisis: Earth and Planetary Science Letters, 2009, vol. 277, no. 3–4, p. 490–500.

[57] Lorenz V., Maars and diatremes of phreatomagmatic origin, a review. Transactions of the Geological Society of South Africa, 1985, 88, p. 459–470.

[58] Lorenz V., Kurszlaukis S., Root zone processes in the phreatomagmatic pipe emplacement model and consequences for the evolution of maar–diatreme volcanoes: Journal of Volcanology and Geothermal Research, 2007, 159, p. 4–32.

[59] Sisson T.W., Grove T.L., Coleman D.S., Hornblende gabbro sill complex at Onion Valley, California, and a mixing origin for the Sierra Nevada batholith: Contributions to Mineralogy and Petrology, 1996, 126, p. 81–108.

[60] Grove T.L., Gerlach D.C., Sando T.W., Origin of calc-alkaline series lavas at Medicine Lake volcano by fractionation, assimilation and mixing: Contributions to Mineralogy and Petrology, 1982, 80, p. 160–182.

[61] Barat F., de Lépinay B.M., Sosson M., Müller C., Baumgartner P.O. and Baumgartner-Mora C., Transition from the Farallon Plate subduction to the collision between South and Central America: Geological evolution of the Panama Isthmus: Tectonophysics, 2014, 622, pp.145–167.

[62] Farris DW. Panama arc magmatism and the evolution of the Canal extensional zone. 2013 GSA Annual Meeting in Denver 2013 Oct 28, Geological Society of America Abstracts with Programs. Vol. 45, No. 7, p. 290.

[63] Fowler, G.D., Geology and geochemistry of the western Panamá Canal basin volcanic arc rocks: Florida State University, Masters Thesis, 2015, 120p.

[64] Mynhier, K.N., Gravity modeling constraints on the Gatun-Chagres basin and tectonic evolution of north-central Panama: Florida State University, Masters Thesis, 2015, 66p.

[65] Ramírez DA, Foster DA, Min K, Montes C, Cardona A, Sadove G. Exhumation of the Panama basement complex and basins: Implications for the closure of the Central American seaway. Geochemistry, Geophysics, Geosystems. 2016 5 1;17(5):1758–77.

[66] Rockwell T.K., Bennett R.A., Gath E., Franceschi P., Unhinging an indenter: A new tectonic model for the internal deformation of Panama: Tectonics, 2010, vol. 29, TC4027,

[67] Pratt T.L., Homles M., Schweig E.S., Gomberg J., and Cowan H.A., High resolution seismic imaging of faults beneath Limon Bay, northern Panama Canal, Republic of Panama: Tectonophysics, 2003, 368, p. 211–227.

